# Transfected *Babesia bovis* Expressing a Tick GST as a Live Vector Vaccine

**DOI:** 10.1371/journal.pntd.0005152

**Published:** 2016-12-02

**Authors:** Daiane P. Oldiges, Jacob M. Laughery, Nelson Junior Tagliari, Ronaldo Viana Leite Filho, William C. Davis, Itabajara da Silva Vaz, Carlos Termignoni, Donald P. Knowles, Carlos E. Suarez

**Affiliations:** 1 Centro de Biotecnologia Universidade Federal do Rio Grande do Sul, Universidade Federal do Rio Grande do Sul, Porto Alegre, Rio Grande do Sul, Brazil; 2 Department of Veterinary Microbiology and Pathology, Washington State University, Pullman, Washington, United States of America; 3 Faculdade de Veterinária Universidade Federal do Rio Grande do Sul; Universidade Federal do Rio Grande do Sul, Porto Alegre, Rio Grande do Sul, Brazil; 4 Departamento de Bioquímica Universidade Federal do Rio Grande do Sul; Universidade Federal do Rio Grande do Sul, Porto Alegre, Rio Grande do Sul, Brazil; 5 Animal Disease Research Unit, Agricultural Research Service, United States Department of Agriculture, Pullman, Washington, United States of America; Instituto Butantan, BRAZIL

## Abstract

The *Rhipicephalus microplus* tick is a notorious blood-feeding ectoparasite of livestock, especially cattle, responsible for massive losses in animal production. It is the main vector for transmission of pathogenic bacteria and parasites, including *Babesia bovis*, an intraerythrocytic apicomplexan protozoan parasite responsible for bovine Babesiosis. This study describes the development and testing of a live *B*. *bovis* vaccine expressing the protective tick antigen glutathione-S-transferase from *Haemaphysalis longicornis* (HlGST). The *B*. *bovis* S74-T3B parasites were electroporated with a plasmid containing the bidirectional *Ef-1α* (*elongation factor 1 alpha*) promoter of *B*. *bovis* controlling expression of two independent genes, the selectable marker *GFP-BSD* (*green fluorescent protein–blasticidin deaminase*), and *HlGST* fused to the *MSA-1* (*merozoite surface antigen 1*) signal peptide from *B*. *bovis*. Electroporation followed by blasticidin selection resulted in the emergence of a mixed *B*. *bovis* transfected line (termed HlGST) in *in vitro* cultures, containing parasites with distinct patterns of insertion of both exogenous genes, either in or outside the *Ef-1α* locus. A *B*. *bovis* clonal line termed HlGST-Cln expressing intracellular GFP and HlGST in the surface of merozoites was then derived from the mixed parasite line HlGST using a fluorescent activated cell sorter. Two independent calf immunization trials were performed via intravenous inoculation of the HlGST-Cln and a previously described control consisting of an irrelevant transfected clonal line of *B*. *bovis* designated GFP-Cln. The control GFP-Cln line contains a copy of the GFP-BSD gene inserted into the *Ef-1α* locus of *B*. *bovis* in an identical fashion as the HIGST-Cln parasites. All animals inoculated with the HlGST-Cln and GFP-Cln transfected parasites developed mild babesiosis. Tick egg fertility and fully engorged female tick weight was reduced significantly in *R*. *microplus* feeding on HlGST-Cln-immunized calves. Collectively, these data show the efficacy of a transfected HlGST-Cln *B*. *bovis* parasite to induce detectable anti-glutathione-S-transferase antibodies and a reduction in tick size and fecundity of *R*. *microplus* feeding in experimentally inoculated animals.

## Author Summary

The cattle tick *Rhipicephalus microplus* is a hematophagous ectoparasite, responsible for the transmission of lethal parasites such as *Babesia sp*, limiting cattle production in tropical and subtropical regions of the world. There is an urgent emerging need for improved methods of control for these currently neglected tick and tick borne diseases. It is hypothesized that a dual attenuated-live vector vaccine containing a stably transfected tick antigen elicits protective immune responses against the parasite and the tick vector in vaccinated cattle. Live *Babesia* vaccines based on attenuated parasites are the only effective method available for preventing acute babesiosis. On the other hand, glutathione-S-transferase from *Haemaphysalis longicornis* (HlGST) is a known effective antigen against *Rhipicephalus microplus*, the most common vector for *B*. *bovis*. This study describes the development and testing of a transfected, *B*. *bovis* vaccine expressing HlGST against the tick *R*. *microplus*. A *B*. *bovis* clonal line designated HlGST-Cln expressing HlGST and GFP/BSD, and separately a control transfected *B*. *bovis* clonal line expressing only GFP/BSD was used to vaccinate calves in two independent experiments. All immunized calves developed mild babesiosis, and only calves immunized with the HlGST-Cln parasite line generated anti-HlGST antibodies. Tick egg fertility and fully engorged female tick weight were reduced significantly in *R*. *microplus* feeding on HlGST-Cln-vaccinated calves. Taken together, these data demonstrates the ability of transfected *B*. *bovis* to elicit antibodies against a heterologous tick antigen in cattle and to induce partial protection in the vaccinated animals against the cattle tick for the first time, representing a step toward the goal to produce a live vector anti-tick vaccine.

## Introduction

The cattle tick *Rhipicephalus microplus* is a hematophagous ectoparasite limiting cattle production in tropical and subtropical regions of the world [1–4]. Methods to lessen the impact of *R*. *microplus* are based almost exclusively on the use of several chemical acaricides, including arsenics, organophosphorus, carbamates, chlorinated hydrocarbons, pyrethroids, macrocyclic lactones and benzoyl phenyl ureas [5]. However, this approach generates undesired consequences such as the selection of acaricide resistant tick populations and contamination of the environment and animal products [6,7]. In this scenario, alternative tick control methods, such as vaccination, are increasingly needed [8,9].

Tick vaccines for the control of cattle tick infestations such as TickGARD and Gavac [10,11] became commercially available in the early 1990's, and are both derived from the *R*. *microplus* midgut membrane-bound recombinant protein Bm86. However, none of the Bm86 derived vaccines are consistently efficient worldwide and the need for new tick vaccines remains [12,13]. Consequently, a growing number of *R*. *microplus* vaccine candidate tick proteins have been identified and evaluated, including Bm86 orthologues and homologs [14–16], tick salivary proteins [17], embryo enzymes [18,19], ribosomal protein [20], and detoxification molecules [21,22], among others.

The glutathione-S-transferases are a class of enzymes involved in detoxification of electrophilic substrates by their conjugation with glutathione [23]. GSTs from distinct species have been investigated as vaccine candidates against several parasites, such as *Necator americanus* [24], *Schistosoma japonicum* [24,25], *Schistosoma mansoni* [26], *Trichinella spiralis* [27], and *Wuchereria bancrofti* [28]. The use of GST in experimental vaccines resulted in variable degrees of protection against the targeted parasites, demonstrating their potential for generating protective immunity [29]. Furthermore, an experimental tick vaccine based on recombinant *Haemaphysalis longicornis* glutathione-S-transferase (HlGST) [30] elicited partially protective responses in bovines against *R*. *microplus* [21,22]. An additional striking and positive feature of HlGST vaccination was an increase in cattle weight gain in comparison to control animals [22].

The impact generated by *R*. *microplus* on cattle health is dual, in part due to a direct effect of attachment and blood ingestion [31], and due to the high morbidity and mortality caused by the numerous pathogens transmitted by this tick, including *Babesia* spp. and *Anaplasma* spp [7,32]. Bovine babesiosis is an acute and chronic disease caused by protozoan parasites of the genus *Babesia*, including *B*. *bovis* and *B*. *bigemina* [33]. If natural exposure to *Babesia* occurs at an early age, cattle normally develop subclinical disease and become immune to subsequent homologous parasite challenge as adults [34]. In contrast, exposure of *Babesia*-naive adult animals usually results in fatal acute disease [35]. Several vaccination procedures based on attenuated strains are available and commonly used as control methods to prevent acute *Babesia* infections in several countries [2,36]. Vaccination with live attenuated *B*. *bovis* strains usually results in mild acute and persistent infections in vaccinated calves, and the elicitation of strong immune responses conferring long-term protection against challenge with homologous and heterologous strains of the parasite [2]. Despite the risk of reversion of virulence, an important safety issue in live vaccines, *B*. *bovis* live attenuated vaccines have now been safely used as field vaccines, without reversion to virulence [37, 38].

Efficient transfection methods, which allow the incorporation and expression of foreign DNA into a parasite host genome, have been developed for *B*. *bovis*, and can also be applied to vaccine development. It was previously proposed that a transfected *B*. *bovis* expressing heterologous parasite proteins can be used as carriers to deliver selected antigens to the bovine immune system [39]. Clearly, transfection methods together with other related gene editing tools allow production of specifically designed strains for developing alternative and better defined attenuated *B*. *bovis* strains [1], and live vector vaccines effective against other parasites [2]. Ideally, such foreign antigen delivery platforms could be applied for developing dual *Babesia* and tick vaccines by producing a *B*. *bovis* strain able to synthetize a tick protein that induces anti-tick immune responses during cattle infection as well as the expected anti-babesia immune response [2,39]. However the ability of transfected *B*. *bovis* parasites to serve as vaccine delivery platforms remains so far an untested approach. This study describes the development and testing of a recombinant *B*. *bovis* strain able to express the tick protein HlGST and its ability to protect against a tick challenge. The results represent a step toward the goal of producing a live vectored anti-tick vaccine.

## Results

### *B*. *bovis* transfection and molecular characterization of transfected parasites

**Fig 1:**
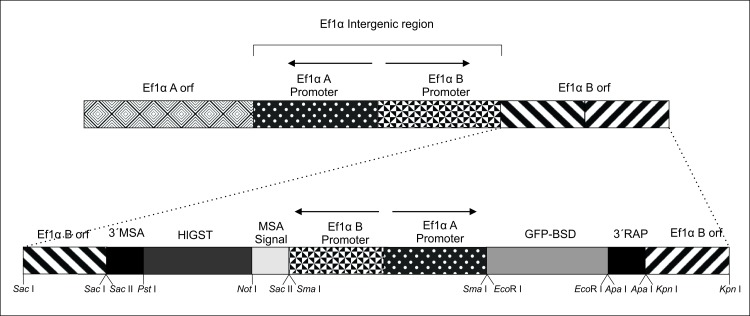
Map of the *Ef-1α* gene structure and the *pMSASignal-HlGST-GFP-BSD* plasmid. The bidirectional promoter and orfs of *Ef1α*-A and B are represented in the upper part of the panel. The dotted lines indicate the targeted site for insertion of the transfected sequences into the genome of the *B*. *bovis*. Arrows indicate the direction of transcription. The location of restriction sites of interest are also described in the figure.

The transfection plasmid *pMSASignal-HlGST-GFP-BSD* is represented in Fig 1. The “B” expression site of plasmid *pMSASignal-HlGST-GFP-BSD* contains a chimeric gene *MSA1-HlGST* encoding a 21 amino acid fragment of the *B*. *bovis* MSA-1 protein corresponding to the signal peptide fused to a 672-bp fragment encoding the 222 amino-acids of the full size HlGST protein of *H*. *longicornis* (Fig 1). Plasmid *pMSASignal-HlGST-GFP-BSD* also includes the *GFP-BSD* selectable marker fusion gene cloned upstream of the *Ef-1α* IG region on the “A” promoter side, and flanking 5′ and 3′ *Ef-1α* ORF sequences to facilitate integration of the two exogenous genes and the bidirectional E*f-1α* promoter into the E*f-1α* locus of the *B*. *bovis* genome [45,49] (Fig 1).

**Fig 2:**
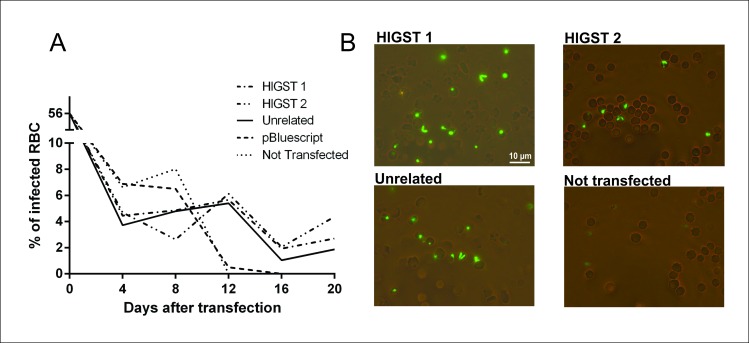
Characterization of transfected parasites. Two lines of transfected parasites HlGST1 and HlGST2 were generated by transfection of the T3B strain of *B*. *bovis* with plasmid *pMSASignal-HlGST-GFP-BSD* and analyzed in these experiments A) Comparison of the growth curves of non-transfected, control transfected (negative control electroporated with plasmid *pBS*, and unrelated positive control electroporated with plasmid *pEf-msa-1-Bm86ep-gfp-bsd*), and two lines of parasites electroporated with *pMSASignal-HlGST-GFP-BSD* (HlGST1 and HlGST2) after electroporation in the presence inhibitory doses of blasticidin. Blasticidin resistant parasites emerge ~16 days after the onset of selection only in the wells containing parasites electroporated with the *pMSASignal-HlGST-GFP-BSD* and *pEf-msa-1-Bm86ep-gfp-bsd* plasmids. B) Fluorescence microscopy of transfected parasites of the HlGST line (HlGST1 and 2, Upper panels), control GFP-*B*. *bovis* line (Unrelated) and non-transfected parasites (Lower panels).

**Fig 3:**
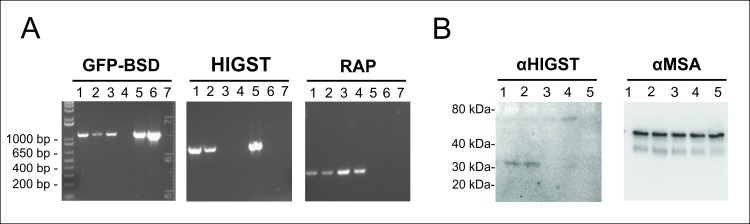
HlGST expression in transfected parasites. A) RT-PCR to detect transcripts of GFP-BSD, GST, and RAP as constitutive control. Lane 1: HlGST1 transfected *B*. *bovis*. Lane 2: HlGST2 transfected *B*. *bovis*. Lane 3: unrelated (GFP) transfected control *B*. *bovis*. Lane 4: non-transfected control *B*. *bovis*. Lane 5: *MSASignal-HlGST-GFP-BSD* plasmid. Lane 6: unrelated transfection control plasmid. Lane 7: negative control. B) Western Blot analysis on transfected parasites using αGST and αMSA-1 antibodies. Lane1: HlGST1 transfected *B*. *bovis*. Lane2: HlGST2 transfected *B*. *bovis*. Lane 3: unrelated transfected control *B*. *bovis*. Lane 4: unrelated (GFP) transfected control *B*. *bovis*. Lane 5: non-transfected control *B*. *bovis*.

*Babesia bovis* T3B parasites were electroporated with plasmids *pMSASignal-HlGST-GFP-BSD*, and control plasmids *pEf-msa-1-Bm86ep-gfp-bsd* [39] or *pBlueScript* (*pBS*). Blasticidin resistant parasites electroporated with plasmid *pEf-msa-Bm86ep-gfp-bsd*, designated Tf-Bm86ep-gfp-bsd, or plasmid *pMSASignal-HlGST-GFP-BSD*, termed HlGST, emerged in *in vitro* cultures starting 16 days after electroporation (Fig 2A). Expression of green fluorescent protein (GFP) was evident upon fluorescence microscopy in both emerging blasticidin-resistant parasite lines (Fig 2B), Transfected fluorescent parasites were also used to verify evasion of parasites from infected RBCs (S1 video). In addition, simultaneous production of the reporter (GFP) and the tick protein (HlGST) by the *pMSASignal-HlGST-GFP-BSD* transfected parasites, termed HlGST, was confirmed by RT-PCR and Western blot analysis (Fig 3A and 3B). The RT-PCR amplifications demonstrated transcription of both *GFP-BSD* and *HlGST* genes in the HlGST parasites maintained in culture (Fig 3A, line 1 and 2). Consistently, *GFP-BSD* but not of *HlGST* transcripts were detected in the transfected control parasite line Tf-Bm86ep-gfp-bsd (Fig 3A, line 3 of GFP-BSD and GFP boxes), and no *GFP-BSD* nor *HlGST* transcripts were detectable in non-transfected, non-blasticicin selected parasites (Fig 3A, line 4 of GFP-BSD and HlGST boxes). Also, *RAP-1* (rhoptry-associated protein 1) transcripts were detected in all parasite lines tested, and no transcripts were detected when transfection plasmids were used as template in the RT-PCR reactions (Fig 3A). Additionally anti-HlGST rabbit antibodies specifically recognize a protein of approximately 30 kDa, a size which is consistent with the predicted size of the MSASignal-HlGST chimera, only in the HlGST transfected parasites in immunoblots (Fig 3B, lines 1 and 2). An approximately 42 kDa band was detected in all *B*. *bovis* culture lysates when the blots were incubated with a control monoclonal antibody against the merozoite surface antigen-1 (MSA-1) from *B*. *bovis* (Fig 3B).

**Fig 4:**
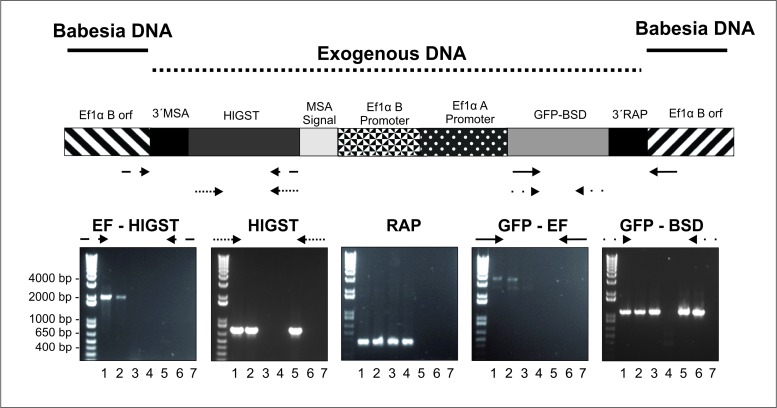
Integration PCR analysis. Upper panel: Representation of the genome area including the transfected genes integrated into the genome of HlGST-Cln *B*. *bovis*. The localization of the regions hybridizing with the primers used in PCR is represented in the map by arrows. Primers were used for the amplification of EF-GST, HlGST, RAP-1, GFP-EF and GFP/BSD. Lower Panel: Agarose gel analysis of the PCR amplification products: Lane 1: HlGST 1 transfected *B*. *bovis* line; lane 2: HlGST 2 transfected *B*. *bovis*; lane 3: unrelated (GFP) transfected control *B*. *bovis*; lane 4: non-transfected *B*. *bovis*: lane 5: *MSASignal-HlGST-GFP-BSD* plasmid; lane 6: unrelated transfection control plasmid; lane 7: negative no DNA control.

Integration into the *Ef-1α* locus was tested by sequencing PCR amplicons derived from HlGST-transfected parasite gDNA. The PCR primers for these experiments were designed to amplify regions that include both exogenous DNA insert and a *B*. *bovis* genomic region lying adjacent to the *Ef-1α* locus (Fig 4, EF-GST and GFP-EF boxes). Identical PCR reactions performed on gDNA from non-transfected *B*. *bovis* or transfection plasmid *pBm86ep-gfp-bsd* did not result in the production of any amplification product. Sequence analysis of the PCR products demonstrated insertion of the foreign transfected genes in the targeted *Ef-1α* locus (GenBank accession number: KX021742).

### Cloning of transfected parasites

**Fig 5:**
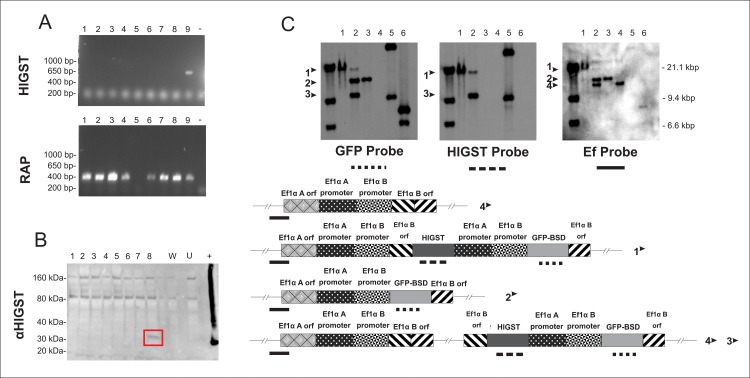
Analysis of the *B*. *bovis* transfected clonal lines. Panel A: RT-PCR amplifications designed for the detection of HlGST and RAP transcripts. A single clonal line (#9) termed HlGST-cln, was able to produce both GST and RAP transcripts. Line 1 to 8: *B*. *bovis* cloned strains, -: negative control, +: positive control. B) Western blot using rabbit serum anti-HlGST to confirm HlGST expression by cloned parasites, confirming the presence of HlGST expression by cell line HlGST-cln (#9) (Red box). Line 1 to 8: cloned *B*. *bovis* strains, W: not transfected parasites, U: unrelated control, +: positive control with recombinant protein produced in *E*. *coli*. C) Southern blot analysis performed on *B*. *bovis* gDNA extracted from HlGST-Cln, HlGST and GFP-Cln *B*. *bovis*. Line 1: GST clonal strain; line 2: GST parent (mixed) population; line 3: GFP control strain; line 4: not transfected parasites; line 5: MSASignal-HlGST-GFP-BSD plasmid; line 6: GFP control plasmid. The arrows marked 1, 2, 3 and 4 represent the distinct hybridizing fragments identified. These fragments are graphically represented in the lower part of the panel C. Each fragment is described in a simplified map of the sequence, and an identifying number on their sides. The parallel bars showed on the sides of each fragment map represent the region digested by *Bgl*II. Lines under the maps in panel C represent the probes used, and the site of binding of the probe on the tested DNA. The dotted line represents the GST probe, the dashed line the GFP-BSD probe, and the continuous line, the EF probe.

Stable transfection experiments using a transfection plasmid containing the *RFP* and e*GFP* genes (*pEf-eGFP-RFP-BSD*, S1 File) using identical plasmid architecture as plasmid *pMSASignal-HlGST-GFP-BSD* (S1A Fig), indicated that the plasmid design used to obtain the transfected HlGST parasites can be stably incorporated into the *Ef-1α* locus of transfected parasites using distinct alternative patterns of insertion. Fluorescence analysis indicates that some of the distinct patterns of insertion preclude the expression of both transfected genes (*GFP* and *RFP-BSD*) by all transfected parasites (S1 Fig). These data confirmed that the stably transfected parasite line HlGST is composed by a mix of parasite subpopulations containing distinct pattern of exogenous gene integration, with some transfected parasites lacking or unable to express the *MSA-1-HlGST* gene. The presence of such a heterologous parasite line composition can interfere with further *in vivo* infection studies, which ideally requires of a homogeneous parasite population expressing both exogenous genes. Based on these observations, the HlGST-transfected culture was then submitted to a cloning procedure using a FACS method [41] in order to obtain a transfected clonal line containing and expressing both, the *GFP-BSD* and *HlGST* genes. Screening of *in vitro* cultures derived from FACS separated cells using a PCR based on the amplification of the *rap-1* gene, identified eight *rap-1* positive culture wells out of the total of 192 wells analyzed (S2 Fig). However, whereas RT-PCR analysis was performed on RNA extracted from the eight *rap-1* positive wells, *rap-1* transcripts were detected in seven of the eight wells (Fig 5A), while HlGST transcripts concurrent with *rap-1* transcripts, were detectable in just a single FACS-separated parasite clonal line (Fig 5A), which was expanded and termed HlGST-Cln. Analysis of Clone 5 was not included on Fig 5B, since the cultured parasites were lost before characterization. Importantly, expression of HlGST in HlGST-Cln parasites was also confirmed by Western blot analysis using anti-HlGST antibodies (Fig 5B). Taken together, these results confirmed the occurrence of a mixed parasite population in the transfected parasite line HlGST which was submitted to FACS sorting, and the ensuing isolation of the clonal line HlGST-Cln able to express the *GFP-BSD* and the *HlGST* genes simultaneously.

Analysis of the pattern of insertion of the transfected *HlGST* and *GFP-BSD* genes in the HlGST-Cln line was performed by Southern blot and PCR. Intact and *Bgl*II digested gDNA extracted from the lines HlGST, HlGST-Cln, GFP-Cln [41] and non-transfected, were analyzed by Southern blots hybridized with *GFP*, *HlGST*, and *Ef-1α* specific dig-labeled probes. The Southern blot data, shown in Fig 5C, indicates that there is only one fragment recognized by all tested probes in the HlGST-Cln line, suggesting the presence of a homogenous parasite population containing a single copy of the exogenous *HlGST* and *GFP-BSD* genes inserted into the expected *Ef-1α* locus. Both GFP and HlGST probes hybridized with the transfection plasmid but did not hybridized with any *Bgl*II digested DNA from non-transfected parasites, confirming the specificity of the probes to the exogenous DNA. However, the GFP probe recognized at least three distinct types of DNA fragments derived from gDNA of the HlGST parasite line. These three distinct patterns of hybridization, named 1, 2, and 3, may be due to the presence of a homogeneous population with multiple insertions, or from a mixed population containing distinct types of insertion. Importantly, the expected 19.3 kb fragment equivalent to the insertion of the exogenous material to the elongation factor region (fragment 1) was also present. Yet, the HlGST labeled probe recognized only two DNA fragments, named 1 and 3, in the *Bgl*II digested gDNA derived from the HlGST parasites (mixed population), suggesting the presence of at least one subpopulation of transfected parasites containing only the *GFP-BSD*, but not the *HlGST* gene.

In addition, an *Ef-1α*-specific probe was also used in order to confirm integration of transfected genes into the expected *Ef-1α-* locus. This probe hybridized with several restriction fragments derived from the HlGST parasite line, designated as 1, 2 and 4 in Fig 5C. Fragment 4 is of the same size as the fragment hybridizing in the non-transfected parasites; fragment 2 has a similar size as the fragment hybridizing with the GFP probe in the clonal line, while fragment 1 is larger than the fragments 2 and 4. Because fragment 1 co-localizes with the single hybridizing fragment of the HlGST-Cln parasite line, it suggests that this DNA is derived from the subpopulation of parasites that integrated the full set of *GFP-BSD* and *HlGST* genes in the expected pattern of integration. Fragment 2 is likely derived from parasites integrating only a part of the exogenous transfected DNA, only the GFP-BSD side of the plasmid. Whilst the presence of parasites lacking the *GFP-BSD* genes is unlikely since all parasites recovered from cloning technique were green fluorescent and resistant to blasticidin, the presence of parasites containing only the reporter/resistance gene occurs, as represented in fragment 2. Regarding fragment 4, it is likely that it might have originated from a subpopulation of parasites with *GFP-BSD* insertions occurring at an alternative site, different than the *Ef-1α* locus, or derived from wild-type parasites still present in the transfected population. Finally, *Bgl*II digested gDNA derived from the GFP-Cln parasites were not recognized by the GST probe, confirming the specificity of the tested probes.

Interestingly, and consistent with previous observations [41], the results collectively, confirmed exclusive stable integration of the transfected genes into the *Ef-1α ORF* gene/locus of *B*. *bovis*. Also, the absence of co-localization of fragments in the same size of the control containing only plasmid DNA confirms the lack of free transfection plasmid or transfection-derived episomal DNA in the HlGST-Cln parasites.

Together, the data confirmed the isolation of a *B*. *bovis* transfected clonal line, termed HlGST-Cln able to express both transfected *GFP-BSD* and *HlGST* genes. Furthermore, the demonstration of co-migrating unique bands with probes EF, GST and GFP in the Southern blots is consistent with a single site of integration of the exogenous transfected genes in HlGST-Cln. We thus conclude that stable insertion of the transfected genes in the genome clonal line HlGST-Cln likely occurred as a single copy in the expected *Ef-1α* locus.

**Fig 6:**
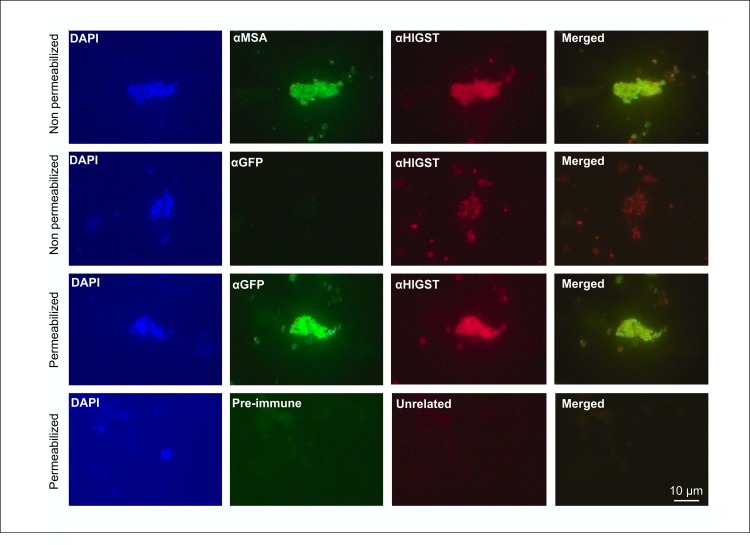
HlGST parasites immunofluorescence. Immunofluorescence assays using DAPI stained permeabilized or non-permeabilized free merozoites derived the from HIGST-Cln *B*. *bovis* cell line. Non-permeabilized free merozoites cells were incubated with anti-MSA-1 (Alexa Fluor 488) and anti-HlGST (Alexa Fluor 555). Non-permeabilized free merozoites were also incubated with anti-GFP (Alexa Fluor 488) and anti-HlGST (Alexa Fluor 555).Permeabilized merozoites were incubated with anti-GFP (Alexa Fluor 488) and anti-GST (Alexa Fluor 555), pre-immune rabbit serum (Alexa fluor 488), control anti-Tryp unrelated (Alexa Fluor 555). Columns represent DAPI, green (488nm), red (555nm) and green/red merged (488nm+555nm). The size bar is indicated on lower right image.

The ability of the clonal line HlGST-Cln to effectively express the HlGST in the external membrane of the transfected *B*. *bovis* merozoites was tested by immunofluorescences (IFA) (Fig 6). The IFA data using non-permeabilized HlGST-Cln free merozoites demonstrates that HlGST, as well as MSA-1, are effectively targeted to the merozoite surface. In contrast, the data strongly suggests that GFP, which lacks a signal peptide, is not localized in the surface layer of the non-permeabilized HlGST-Cln merozoites by specific antibodies (Fig 6).

Collectively, these data indicates that the HlGST-Cln line is an appropriate candidate for testing whether transfected parasites are able to cause acute and persistent infection in bovines while eliciting antibody responses against the HlGST protein.

### Bovine immunization and tick challenge

**Fig 7:**
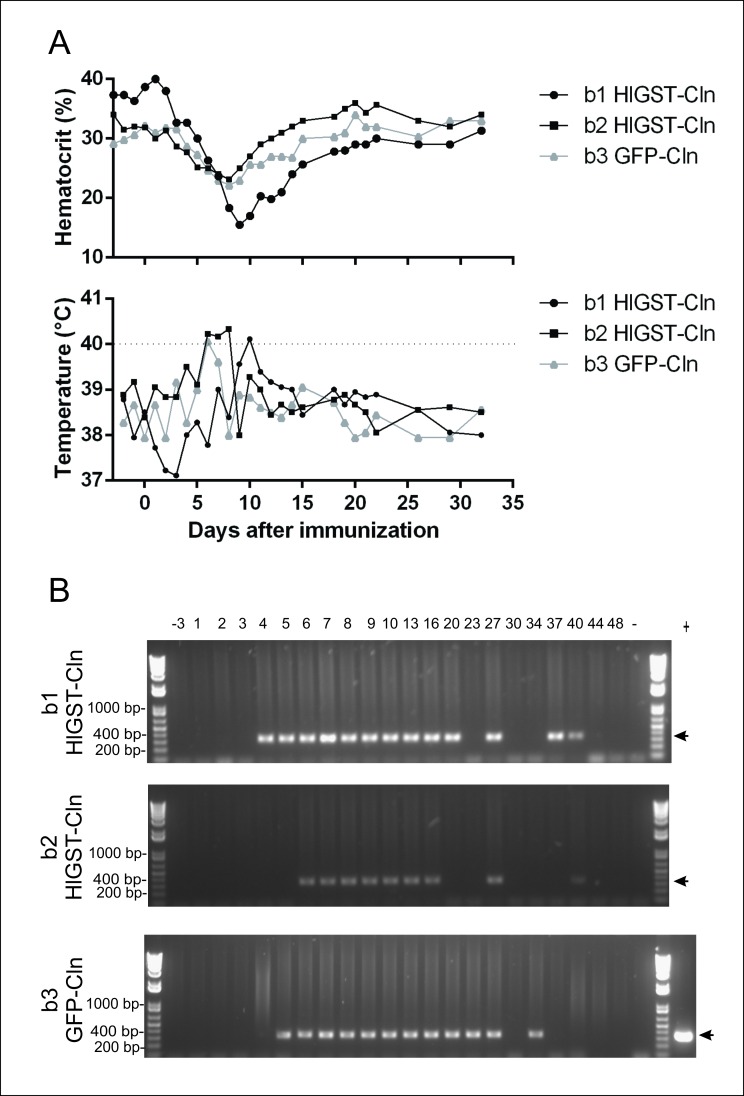
Infection of animals with clonal parasites. Panel A: Daily clinical parameters (PCV and Rectal temperature) of the experimentally infected calves b1, b2 and b3. The dotted line in temperature graphic represents the threshold that indicate fever. Panel B: RAP-1 PCR amplification performed on daily total gDNA samples generated from blood of calves b1, b2 and b3. The expected 387 bp PCR fragment of the *rap-1* gene using DNA isolated from washed RBC of infected animals is marked by arrows. The numbering over the lanes represent the day of blood collection after animals immunization. Size markers are shown on the left ends of the figures.

Two independent immunizations were performed. The first experiment was aimed to demonstrate that infection of cattle with the HlGST-Cln parasite lines cause acute and persistent infection, remaining genetically stable, and elicit antibodies reactive with recombinant HlGST. In this experiment, two calves were experimentally infected with 5×10^7^ infected erythrocytes of the parasite HlGST-Cln line (calves b1 and b2) and one control animal was experimentally infected with the same amount of *B*. *bovis* T3B-derived clonal line GFP-Cln parasites [41] (calf b3). All animals presented an increase in rectal temperatures above 40°C at some point during the acute stage of the disease, and reduction in hematocrit 7 days after immunization (Fig 7A). The presence of *B*. *bovis* in the blood of experimentally infected animals was confirmed by PCR (Fig 7B). While PCR revealed the presence of circulating merozoites in the blood in all animals (Fig 7B), no parasites were visualized in blood smears from jugular blood samples. Overall, these data suggests that all three experimentally infected calves developed similar clinical symptoms of mild babesiosis.

Both *B*. *bovis* strains used in immunization were culture-recovered from the blood of animals 8 days after immunization and analyzed (S3 Fig). Blasticidin-resistant fluorescent parasites were detected 10 days after the establishment of the *in vitro* cultures from all animals. RT-PCR, gDNA PCR, western and southern blot were performed with the recovered parasites, showing that the recovered HlGST-Cln-recovered parasites remain genetically stable, retain the ability to express the GFP-BSD and HlGST genes, and the clonal characteristic of cell lineages (S3 Fig), and thus they appear to be similar to the inoculated HlGST-Cln parasites.

**Fig 8:**
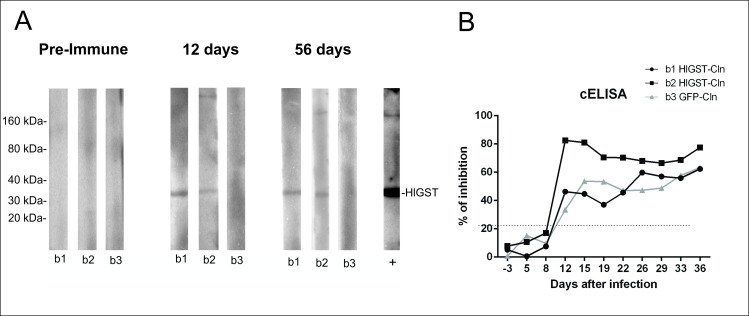
Detection of antibodies in calves experimentally infected with HlGST-Cln and GFP-cln parasites by cELISA and western blot analysis. (A): Western blot analysis of recombinant HlGST incubated pre-immune and immune (12 and 56 days post immunization) sera from calves’ b1, b2, and b3 diluted 1:10. +: positive control, recombinant HlGST incubated with anti-HlGST serum, in a 1:1000 dilution. (B) Kinetics of antibody detection of the rhoptry-associated protein 1 (RAP-1) of *B*. *bovis* by cELISA. Samples obtained from each animal before and 10 days after experimental intravenous inoculation of the parasites. The threshold of inhibition is 21%, above which samples are considered positive is represented by the dashed line.

Serological detection of anti-HlGST antibodies was performed using bovine sera from vaccinated and control groups. Western blot analysis show the specific recognition of recombinant HlGST by antibodies in the bovine sera from both calves (b1 and b2) experimentally inoculated with the HlGST-Cln line (Fig 8A) beginning at day 12 post-inoculation, at a 1:10 dilution which was verified until day 56 post-inoculation. Presence of antibodies reactive with HlGST confirmed expression of the transfected protein during the infection. In addition, the production of antibodies against RAP-1 was also determined routinely for each animal using a cELISA [47,48]. Anti-RAP-1 antibodies were also detected starting at 12 days post-inoculation (Fig 8B).

Once it was demonstrated that the transfected parasites were able to elicit mild acute and persistent infection, remain genetically stable, and generate anti-HlGST antibodies, we investigated whether calves infected with the parasite line HlGST-cln are able to interfere with tick development upon tick challenge in a separate experiment. To test this, we infected a group of three age-matched Hereford calves with the transfected strain HlGST-Cln (animals B1, B2 and B3) and three age-matched calves with the GFP-Cln transfected control strain (animals B4, B5 and B6). All infected animals received a 5x10^7^ parasite inoculum. Similar as in the previously described immunization experiment, hematocrit and the rectal temperature were measured every day during the first 10 days after immunization. All animals had a gradual reduction in hematocrit after immunization, a clinical characteristic signal of acute babesiosis (S4A Fig). Rectal temperatures were measured in the same period and according to the threshold for fever determination, only one of the six animals showed increased rectal temperature above 40°C (S4B Fig). Interestingly, upon comparison to the 3 Holstein calves used in the first immunization experiment, the 6 Hereford calves of the vaccination trial presented a lighter response to *B*. *bovis* infection. Fibrinogen, an important acute phase protein [50], was also measured daily during the 10 days after immunization as an indicator for the presence of acute infection. All animals presented significant increase in fibrinogen levels during days 3 and 4 (p<0.01) post-infection, compared with pre-vaccination levels (S4C Fig), but fibrinogen levels were reduced thereafter.

The six animals used in the vaccination trial experiment were also subjected to additional biochemical and hematologic exams prior to vaccination and 5 and 10 days after vaccination. This intensive clinical following was done in order to check the possibility of other *Babesia*-unrelated clinical conditions in the animals subjected to vaccination with the recombinant parasite. Urea, creatinine, aspartate aminotransferase, alkaline phosphatase, albumin, total protein and total globulin were tested using immunized animals serum. None of those assays presented a significant clinical change after vaccination (S1 Table). For hematological parameters a reduction in total leukocytes was verified in day 5 after immunization (P<0.05), but all animals already recovered at day 10 after immunization and by then, their leukocyte levels were indistinguishable from the levels prior to immunization (P>0.05) (S2 Table). Despite total leukocyte reduction, it was not possible to evaluate any specific reduction among neutrophils, lymphocytes, monocytes, eosinophils or basophils (Repeated measures ANOVA P>0.05). All hematologic values are described in S2 Table. Importantly, even with the reduction at day 5, the leukocyte total counting stayed at levels considered similar to the normal reference value determined for bovines (S2 Table).

Serological analysis on the HlGST-Cln vaccinated animals showed presence of detectable anti-HlGST antibodies in immunoblot analysis, corroborating with the previous immunization experiment (S5 Fig). All experimental animals were subjected to a tick challenge for further collection of engorged females thirty days after immunization. Evaluation in the number and weight of fully engorged females demonstrated a significant reduction in individual tick weight among ticks derived from the three animals experimentally infected with the HlGST-Cln line (p<0.05) (Table 1), even though no difference in total weight or tick number was detected (p>0.05) (Table 1). In addition, egg fertility was reduced in ticks obtained from the calves vaccinated with transfected parasites expressing HlGST (P<0.05) (Table 1) compared with the GFP-control group.

**Table 1:** Biological parameter of detached *R*. *microplus* from vaccinated and control cattle groups

		Fully engorged females^a^			Index	
	Animal	Number	Weight (g)	Individual weight (g)	Eggs laying capacity^b^	Eggs fertility^c^
**HlGST-Cln**	B1	1443.00	270.53	0.25	0.47	0.33
	B2	2051.00	468.77	0.27	0.41	0.31
	B3	2329.00	549.26	0.26	0.45	0.33
Total		5823.00	1288.56	0.79	1.33	0.97
Mean		1941.00	429.52	0.26	0.44	0.32
SEM		261.61	82.82	0.008	0.017	0.005
**GFP-Cln**	B4	4276.00	1156.20	0.30	0.38	0.37
	B5	1571.00	366.61	0.30	0.46	0.37
	B6	658.00	79.48	0.29	0.44	0.35
Total		6505.00	1602.29	0.90	1.29	1.09
Mean		2168.33	534.09	0.30	0.43	0.36
SEM		1086.29	321.90	0.003	0.024	0.007
**Difference**^**d**^		10.484	19.580	12.389 *	-3.333	10.593*

SEM: standard error of mean

^a^ Female ticks detached during infestation period.

^b^The eggs weight laid by sample of fully engorged tick during infestation period was used to calculate the proportion of the weight of ticks that was converted into eggs, named egg laying capacity.

^c^ Eggs fertility represent the laid eggs converted into larvae.

^d^ Difference (%) = 100 × (1 − mean value of vaccinated group/control group).

*Statistically significant (p < 0.05).

## Discussion

The *B*. *bovis* protozoan presents a highly complex life cycle that includes a bovine host and *Rhipicephalus microplus* tick vector. The ability of the tick to perform a transovarian transmission to the new generation represents an effective mechanism for babesial dissemination and reinforces the critical role of the tick as a vector. As a result, efficient tick vector control is an essential strategy for eradication of this disease [51].

Live vector vaccine approaches are well described using simple organisms such as virus and bacteria as delivery platforms [52–56]. This vaccine methodology has the potential advantage of presenting foreign antigen to the immune system in the context of an infection, which can induce a better immune response, and also be able to amplify the stimuli due to organism multiplication, which is different and potentially more effective than subunit vaccine approaches [56]. However, few studies using eukaryotes as live vectors are currently available in the literature. These include the use of *Toxoplasma gondii* as a live vaccine vector against *Eimeria tenella* infection in chickens [57], transgenic *Leishmania tarentolae* against the pathogenic strains *L*. *donovani* and *L*. *infantum* [58], and a construction of *Neospora caninum* stably expressing a *T gondii* protein for further evaluation of its protective effects against *T*. *gondii* infection in mice [59].

A limiting step to achieve an efficient system for delivery of heterologous antigens via a recombinant live vector is the availability of a genetic modification tool that permits the modification of the desired vectors, such as transfection. For apicomplexan parasites as *Plasmodium sp* and *Toxoplasma sp* efficient transfection and gene editing methods have been developed [60–64]. Unfortunately, much less progress has been achieved for the genetic manipulation of *B*. *bovis*. Overcoming our limited ability to genetically manipulate this organism is vital to the better understanding of the biology of this parasite. A *B*. *bovis* transfection system was previously developed and can be useful for both vaccine development and study of the parasite biology. As shown in the S1 Video, fluorescent transfected parasites of the line HlGST allow direct visualization of specific parasite mechanisms of interest such as infected red blood cell lysis, and probably erythrocyte invasion, as previously showed [65], using fluorescent microscopy techniques. In addition, transfection techniques can be instrumental for developing novel vaccine approaches, including the development of a vaccine delivery system based on transfected *Babesia* parasites. Ideally, such vaccines should be designed to contain a homogeneous population of parasites able to express a heterologous antigen of interest during the natural course of infection. Also, the gene coding for the heterologous antigen of interest should remain stably integrated to the genome of the vector parasite even after several replication cycles of the vaccine vector in the infected host.

It is also important to determine whether transfection results in fitness cost to the parasite. In previous papers [41] it was showed that the transfection targeting the *B*. *bovis ef -1α* locus, such as performed in this study, do not alter the growth of parasites compared to the non-transfected control T3Bo parasites. In addition, *in vivo* infection studies comparing such transfected *vs* non-transfected parental parasites, [66] suggested the lack of apparent fitness costs to the parasite. These studies concluded that transfected parasites are genetically stable, and possess the characteristics required for a recombinant attenuated *B*. *bovis* vaccine.

Transfection of plasmid *pMSASignal-HlGST-GFP-BSD* into S74-T3B *B*. *bovis* parasites resulted in the stable integration of exogenous genes into the genome of the parasites. This plasmid was designed for the insertion into the *Ef-1α* locus and for the expression of a chimera version of the HlGST gene driven by the *Ef-1α* promoter “B”. The chimera gene included the signal peptide of the *B*. *bovis* MSA-1 fused to the full size *HlGST* orf. This fragment coding for the MSA-1 signal peptide was added to the 5’ region of the gene coding for HlGST in order to facilitate surface expression of the protein, as previously demonstrated [39] a configuration likely resulting in improved immunogenicity.

The transfected plasmid encoding for HlGST was able to successfully integrate into the *B*. *bovis* genome. However, and likely as a result of the complexity of the transfection construct containing regions that can facilitate homologous recombination, not all transfected parasites have the same integration profile and not all of them were able to express both transfected proteins simultaneously, which became clearly evident when a similar dual fluorescent construction was tested (S1 Fig). This observation is relevant to vaccine development since, as mentioned before, ideally a live vaccine should be based on a single homogeneous population in order to avoid the possible occurrence of selection mechanisms for non-vaccine relevant parasite subpopulation during infection [67]. In this scenario, all parasites of the vaccine strain should also be able to constitutively express the antigen of interest during the infection in order to maximize antigen exposure to the immune system. In preliminary experiments using parasites transfected with the dual promoter controlling expression of the *RFP-BSD* and *eGFP* genes (S1 Fig), we found a majority of parasites growing in *in vitro* cultures selected with blasticidin only expressing RFP, likely because the plasmid can insert in the genome in alternative patterns, and the *RFP* gene is linked to the blasticidin resistance gene. Interestingly, this data is consistent with the genetic and expression analyses of several clonal lines derived from the HlGST transfected parasite lines indicating that a great proportion of the transfected parasites did not present insertion of the *HlGST* ORF and consequently were unable to express the heterologous protein, and thus, irrelevant components for a vaccine based on transfected parasites expressing heterologous antigens. Together, these findings emphasize the need for further parasite selection following transfection and blasticidin selection using parasite cloning methods.

The availability of a clonal line expressing both, the GFP-BSD and HlGST proteins allowed *in vivo* infection in bovines. The initial experimental infections study performed in Holstein calves showed that the HlGST-Cln parasites are able to cause mild acute and persistent infections in the bovine host, both desirable attributes of a live vaccine. Analysis of recovered parasites demonstrated that these parasites remained genetically stable, and able to express the heterologous protein. Importantly, the HlGST protein generated by the transfected parasites during infection was able to elicit humoral immune responses that recognize the recombinant HlGST protein. Thus, the data obtained in the first *in vivo* experiments supported further testing of the experimental vaccine using a larger number of animals, and followed by tick challenge after immunization.

The second experimental immunization study included, in addition to the traditional hematocrit and temperature measurements, a more intensive and multifactorial panel of clinical studies, in order to verify if animals subjected to vaccination with parasites of the HlGST-Cln line developed additional clinical alterations. All infected animals presented the classical signs of babesiosis (temperature increase and hematocrit reduction) but none of the animals were prostrated and only one of them presented a temperature above of the threshold considered as fever. Consistent with the previous experiment involving Holstein calves, the Hereford calves also develop mild disease upon infection, but to a lesser degree. Difference in babesiosis susceptibility is well characterized between *Bos taurus taurus* and *Bos taurus indicus* cattle, the former being more susceptible to babesiosis [68]. It is also known that there are differences in the response to *Babesia* infection among cattle belonging to distinct *Bos taurus* breeds [69], which could be responsible for the differences observed in the response to infection among the two groups of animals tested in these studies. Taken the data of the second bovine trial together, none of the infected animals presented alterations in the biochemical parameters measured in the study (urea, creatinine, *etc*.) suggesting that vaccination with transfected parasites did not compromise the overall fitness of vaccinated calves.

Calves experimentally infected with the HlGST-cln parasites in both immunization experiments developed relatively low antibody titers against HlGST, and both serum presented recognition of recombinant HlGST only at a 1:10 dilution. However, the second vaccination experiment also demonstrated anti-tick activity for ticks feeding in vaccinated animals. These data indicates that the humoral response against HlGST expressed by transfected parasites was relatively weak and markedly lower in comparison with the response generated by animals immunized with recombinant protein in previous investigations [21,22]. This outcome is similar to the findings described by Zou et al [57] that used an engineered strain of *T*. *gondii* designed to express the yellow fluorescent protein (YFP) in the cytoplasm in order to test protection of vaccinated chickens against another engineered pathogen, a strain of *E*. *tenella* also expressing YFP [57]. They report that animals immunized with the transgenic apicomplexan also developed a partial protection, but anti-YFP antibody titers in chickens immunized with the transgenic parasites were markedly lower than those in animals immunized with recombinant YFP protein [57]. At least for the HlGST transfected *B*. *bovis* parasites, it is not possible to discard the possibility that the low humoral response was due to reduced levels of expression of HlGST during infection, which could be related to the transfection plasmid design used in this study.

In this work, A DNA fragment coding for the MSA-1 signal peptide was added to the 5’ region of the gene coding for HlGST in order to facilitate surface expression of the protein, as previously demonstrated [39]. However, despite the confirmed expression of the HlGST in the surface of the transfected parasites of the clonal line in IFA experiments, poor immunogenicity was observed in our study. Collectively, these data suggests that surface exposure of the exogenous antigen might be a necessary but not per se a sufficient requirement for increased antigenicity. Consistently, other previous work using a live vector vaccine approach with a trypanosomatid-based delivery system [72] also showed that externalization of the antigen of interest in the outer membrane of the parasite was not sufficient to induce a strong humoral response. Only when the fusion of the antigen to the N-terminus of a protein responsible for extracellular secretion was done it was possible to see an increased humoral response. It is also possible that regulation of the expression of the *ef-1α* B promoter is different among cultured and *in vivo* developed parasites, but testing this possibility was beyond the scope of our study. Alternative solutions to this potential limitation include the use of alternative stronger blood stage promoters, and/or the use of high-gene copy-number expression plasmids. However, the latter approach might be difficult to achieve since larger DNA inserts might be unstable and can potentially compromise the overall fitness of the live vector [70]. Alternatively, it is also possible to target expression of HlGST on the surface of infected erythrocyte, rather than in the merozoite surface. This mode of presentation could continuously potentially expose the antigen to the immune system and subsequently induce stronger immune responses. The *B*. *bovis* variant erythrocyte surface antigen (VESA) is known to be exported to the external membrane of erythrocyte [71]. However, further analysis of the mechanisms used by this protein for erythrocyte surface exposure is needed in order to test this alternative strategy in the transfected antigen of interest. Finally, another possible alternative is targeting secretion of desired antigen to the extracellular milieu [72], however further analysis of the mechanisms used for protein secretion in *B*. *bovis* are also necessary before this approach can be tested.

Remarkably, and despite the presence of relatively low amounts of anti HlGST antibodies, the animals immunized with HlGST-cln parasites in the second vaccination experiment presented a statistically significant reduction in egg fertility and in individual fully engorged female tick weight in comparison with GFP immunized control animals upon challenge with tick larvae. In contrast, it was previously found that vaccination of cattle with recombinant GST [21,22] resulted in a strong anti HlGST humoral immune response and effective protection likely due to a drastic reduction in the amount of eggs produced in ticks feeding on immunized animals. Although vaccination using these two procedures is based on a similar subunit antigen approach, they use different delivery strategies, which may result in dramatic differences in the outcomes upon tick challenge [73]. These differences include conformation of the antigen, the amounts and timing of antigen delivered, adjuvant effects, the possible involvement of different population of antigen-presenting cells, etc. Several vaccines work effectively through eliciting antibodies in serum or on mucosa in order to induce protection, and consequently the presence of antibodies correlates with effective infection blocking. However protective outcomes not only depend on the quantity of antibodies, but also of its functional characteristics [74] which can be influenced by the method of delivery and antigen presentation mechanisms.

Another hypothesis that should be tested in the future is the use of a *Babesia* based live vector vaccine as a dual vaccine. However, the focus of this study was limited to the development of an anti-tick vaccine, and consequently the ability of this vaccine to protect against further *B*. *bovis* challenge was not analyzed. In order to exploit this dual vaccine characteristic, further transfection assays should be done using *B*. *bovis* attenuated strains, such the ones used in live vaccines formulations.

In summary, we described a transfected *B*. *bovis* strain able to express HlGST, a previously demonstrated protective tick antigen that elicits immune responses in the bovine host. Also, we demonstrated that vaccination of calves with the recombinant vaccine caused mild acute disease and did not compromise their general fitness. However, vaccination with HlGST resulted in weak antibody responses against HlGST. Importantly, the vaccine was able to interfere with the life cycle of the tick vectors feeding in the vaccinated animals despite of low HlGST antibody titers. Regardless of the comparisons among recombinant and vectored antigen presentation, this work suggests that the hemoprotozoan *B*. *bovis* can be used as a live vector, but its ability to elicit strong humoral responses against the target antigen needs to be improved [75]. In addition, the design of transfection plasmids should be optimized for unambiguous insertion of the transfected genes into the genome.

In conclusion, these experiments provided important information as the basis to guide further transfection plasmid construction in order to obtain a more fitted and antigenic transfected parasite to be used in a dual live vector vaccine against *B*. *bovis*, ticks or even distinct parasites.

## Materials and Methods

### Parasites

*B*. *bovis* strain S74-T3B [40] and T3B-derived clonal line Tf-149-6 C6 [41] hereby renamed as GFP-Cln, were maintained as a cryopreserved stabilate in liquid nitrogen. Parasites were grown in long term at a stationary phase culture using 10% of bovine red blood cells (RBC) in HL-1 medium supplemented with bovine serum as described by Levy [42] and maintained at 37°C and 5% CO_2_.

### Plasmid constructions

The transfection plasmid *pEf-msa-1-Bm86ep-gfp-bsd* described by Laughery et al [39], was used as a backbone to construct the *pMSASignal-HlGST* plasmid for stable transfection. The *Sac*II restriction fragment of plasmid *pEf-msa-1-Bm86ep-gfp-bsd* containing the MSA1-BM86 chimera gene was removed by restriction enzyme digestion with *Sac*II and replaced by a DNA fragment coding for the *MSASignal-HlGST* fusion gene.

For *MSASignal-HlGST* insert construction the sequence of the *B*. *bovis MSAI* signal peptide containing the restriction sites *Bam*HI (Invitrogen), *Not*I (Invitrogen) and *Sac*II (Invitrogen) was designed, synthesized (Integrated DNA Technologies) and amplified with primers described in Table 2 (MSA-SigBam F and MSA-SigNot/Eco R). The amplicon was cloned into pCR 2.1-TOPO (Thermo Fisher Scientific) cloning vector. The MSASignal fragment cloned in pCR TOPO 2.1 was digested with *Bam*HI (Invitrogen), purified and then ligated in plasmid *pBlueScript* (*pBS*) vector previously digested with same enzymes, and named *pBlue-MSA* plasmid. The *HlGST* sequence was amplified using HlGST-BamHI-SacII F and HlGST-PstI R primers (Table 2) using the plasmid *pET43a-HlGST* [30] as template. This PCR product was cloned in pCR TOPO 2.1, and termed *p2*.*1-HlGST* plasmid. *p2*.*1-HlGST* was digested with *Sac*II (Invitrogen) and *Pst*I (Invitrogen) yielding a restriction fragment containing the *HlGST* gene, which was ligated into the plasmid *pBlue-MSASig* previously digested with the same restriction enzymes. The resulting plasmid, termed *pBlue-MSASig-HlGST* was then digested with *Not*I (Invitrogen) and *Pst*I (Invitrogen) for ligation into the backbone transfection plasmid *pEf-msa-1-Bm86ep-gfp-bsd*, also digested with the same enzymes. All constructs prepared during these steps were sequenced in order to assure the absence of mutations. The final plasmid obtained was designated *pMSASignal-HlGST-GFP-BSD*, and is represented in Fig 1. Plasmid *pMSASignal-HlGST-GFP-BSD* was purified using Plasmid Plus Maxi Columns (Qiagen) for transfections.

**Table 2:** Primers and MSA Signal Peptide template used in plasmid construction

	Name	Sequences	Size (bp)
**Template**			
	MSA Signal Peptide Sequence	gcctagggatccgcggccgcatggctacgtttgctcttttcatttcagccttg tgctgtgttttggcaattacatcggcgggtgaaccgcggggatcctgagac	104
**Primers**			
	MSASigBam F	gcctagggatccttaaaaactaatggtagtgac	33
	MSASigNot/Eco R	gtctcagcggccgcgaattcttatttattaatgttcc	37
	HlGST-BamHI/SacII F	gcgtaaggatccccgcggatggctcctattctcggctac	39
	HlGST-PstI R	cgatcactgcagcggcttcttctgtagcctgctgcc	36
	Tracer-EcoRV-gfp-F	cgtcgtgatatcatggcctccaaaggagaac	31
	EcoRV-bsd-R	taatgtgatatcgccctcccacacataaccagag	34
	Ef-Pr F8	gtctttataacttaataaagtaattcc	27
	UPS-Ef-probe-R	cacgcgcaatatcacagttccatc	24
	BoN-F	tgttcctgagccgctatctt	20
	BoN-R	cagcccatttcacaggtttt	20

### Stable transfection

Plasmid *pMSASignal-HlGST-GFP-BSD* together with control plasmids *pBlueScript* and *pEf-msa-1-Bm86ep-gfp-bsd* [39] were used for transfections. Twenty μg of each plasmid were suspended in 25 μL of cytomix buffer (120 mM KCl, 0.15 mM CaCl_2_, 10 mM K_2_HPO_4_/KH_2_PO_4_, pH 7.6, 25 mM Hepes, 2 mM EGTA, and 5 mM MgCl_2_, final pH 7.6). Parasites were obtained from a flask expansion. The infected red blood cells (iRBC) were centrifuged at 500 g for 5 min to sediment the cells that were washed once in cold filter sterilized cytomix buffer. The final washed cell pellet was re-suspended in volume/volume of cytomix solution to be further added to plasmid. Electroporation was performed in a Gene PulserII apparatus (Bio-Rad) using 0.2 cm cuvettes containing the plasmid/iRBC/cytomix solution, and settings used were 1.2 kV, 200 Ω and 25 fixed capacitance [43,44]. 20 μg of plasmid were suspended in 25 μL of cytomix buffer and electroporated with 75μl of bovine iRBC with a 56% parasitemia.

Following electroporation, iRBC were incubated in 24 well plates containing 1 mL of culture medium and 100 μL of RBC. Four hours after electroporation the medium was changed and selective agent, blasticidin (Invitrogen), added to a final concentration of 4μg/mL. Parasitemia was checked, twice a week, by counting of Diff-Quik (Dade Behring) stained blood smear slides in an optic microscope as described by Suarez and McElwain [44].

### Genetic analysis

Genomic DNA of transfected and control parasites was obtained from cultured parasites as described [45] and used as template for PCR assays designed for analysis of the insertion pattern of the foreign transfected genes into the *B*. *bovis ef-1α* locus (integration PCR). A PCR designed to determine the pattern of transfected sequences into the *B*. *bovis ef-1α* locus was performed using two pairs of primers: the first Ef-Pr F8 + GST-BamHI-SacII F and the second, UPS-Ef-probe-R + Tracer-EcoRV-gfp-F (Table 2). Both forward primers anneal in a sequence originally present in the plasmid used for transfection, and both reverse primers anneal in a *B*. *bovis* genome region located in the vicinity of the *B*. *bovis Ef-1α* locus. Amplification of GFP/GST ORF was performed with primers Tracer-gfp-EcoIF and EcoRV-bsd-R and HlGST was amplified using GST-BamHI-SacII F and GST-PstI R primers (Table 2). Amplification of RAP-1 transcript was used as a control for presence of gDNA and performed with primers BoNF and BoNR (Table 2). PCR products were analyzed in 1% agarose gels and cloned in Topo 2.1 vector (Invitrogen) for posterior sequencing.

Genomic DNA was also used for southern blot analysis. Digoxigenin-labeled probes representing the *HlGST* ORF (GST Probe), the *GFP* ORF (GFP probe) and a 300 bp region upstream of the *Ef-1α* locus (EF Probe), were prepared by PCR amplification using a PCR Dig-Probe Synthesis kit (Boehringer–Roche). The GST probe was prepared by PCR with GST-BamHI-SacII F and GST-PstI R primers (Table 2) using the *pHlGST-pET43* plasmid as template. The EF and GFP-BSD probes were prepared as described by Suarez and McElwain [45]. Total DNA from *B*. *bovis* merozoites was digested with *Bgl*II, electrophoresed during 16h at 20V, capillary transferred to ZetaProbe nylon membranes (Bio-Rad) and hybridized with dig-labeled GST, GFP and EF probes, as previously described by Suarez and McElwain [45]. *Bgl*II do not the casset inserted into babesia genome. The gDNA extracted from a previously described *B*. *bovis* T3B-derived clonal line TF-149-6 C6 [41], and redenominated GFP-Cln in this work, and plasmid DNA obtained from *pMSASignal-HlGST-GFP-BSD* and *pGFP/BSD/Ef* (the plasmid used in TF-149-6 transfection) were all used as controls in the Southern blots.

### Expression analysis

Expression of the reporter gene *GFP* was analyzed by fluorescence analysis using a Zeiss Axioskop fluorescent microscope (Carl Zeiss Micro Imaging) on *in vitro* cultured transfected parasite as previously described [45].

Fluorescent parasites were analyzed by RT-PCR to check for the presence of *GST*, *GFP-BSD* and *RAP-1* transcripts. *B*. *bovis* merozoite total RNA was extracted from *in vitro* cultures by the standard TRIzol (Life Technologies) procedure as described previously [46], and treated with RNAse-free DNAse (Ambion). cDNA was generated using the Superscript First-Strand Synthesis System kit (Invitrogen) from 1 μg of total RNA. A fragment of the *GFP/BSD* ORF transcript was amplified from the cDNA either with the primers Tracer-gfp-EcoIF and EcoRV-bsd-R and the *GST* transcript was amplified using GST-BamHI-SacII F and GST-PstI R primers (Table 2). Amplification of *RAP-1* transcript, used as a wild-type and parasite-derived constitutive control, was performed with primers BoN-F and BoN-R [47] (Table 2). Products of RT-PCR were cloned into vector pCR TOPO 2.1 (Invitrogen) and sequenced.

Protein expression was determined by Western blot analysis using whole culture lysates as previously described [46]. Equal amounts of protein (5 μg) were applied per lane in a 4–20% pre packed gel (Bio-Rad) and submitted to SDS-PAGE. Immunoblots were developed in a nitrocellulose membrane with anti-HlGST rabbit serum at a dilution of 1:1,000, anti-GFP antibody (Invitrogen) at a dilution of 1:1,000 and goat anti-rabbit-immunoglobulin peroxidase conjugate (Life Biosciences). The anti-MSA1 monoclonal antibody BABB35 [39] was used as a positive control for the immunoblots at a concentration of 2μg/ml. Purified recombinant protein produced from *pET43a-HlGST* [30] was used for anti HlGST antibody production. One rabbit was inoculated four times at 15 days intervals by subcutaneous route with 100 μg of recombinant protein. Protein concentration was determined according to the Bradford technique.

Immunofluorescence of extraerythrocytic merozoites was performed using the HlGST clonal line. Merozoites were isolated from HIGST-Cln parasite line with parasitemia over 30% by centrifugation two times at 400 RCF to remove the RBC with a final centrifugation at 2,000 RCF to pellet the merozoites and washed in 3% bovine serum albumin (BSA) PBS. Half of the isolated merozoites were then fixed for 10 minutes using 100% acetone and permeabilized by incubation with Triton X-100 0.1%. The remaining free non-permeabilized merozoites were incubated in 10% BSA with a combination of either 1) anti-GST (1/500) and anti-MSA-1 (mAb BABB35) (7μg/ml) and 2) anti-GST (1/500) for one hour. The cells were then washed in PBS two times with a 400 RCF centrifugation and incubated with 1:1000 10% BSA dilutions of either 1) goat-anti-rabbit Alexa Fluor 555 and goat-anti-mouse Alexa Fluor 488 and 2) goat-anti-rabbit Alexa Fluor 555 and anti-GFP conjugated with Alexa Fluor 488 for one hour. The cells were again washed two times, dried to a slide and mounted with Prolong Gold anti-fade with DAPI. The slides with permeabilized cells were incubated with either 1) anti-GST or 2) pre-immune rabbit, anti-Tryp, a non-relevant monoclonal antibody, for one hour, washed two times in PBS, and then incubated with 1) goat-anti-rabbit Alexa Fluor 555 and anti-GFP conjugated with Alexa Fluor 488, or 2) goat-anti-mouse Alexa Fluor 488 and goat-anti-mouse Alexa Fluor for one hour. All slides were then analyzed with epifluorescence microscopy to produce merged images.

### Cloning of *B*. *bovis* transfected parasites

Flow cytometry was used to obtain a clonal line as described previously [41]. Briefly, 50 μL of a growing culture with 9% PPE was washed once in culture medium and diluted in medium to obtain a cell density suitable for single cell sorting with a FACSVantage cell sorter (Becton-Dickinson) with Diva Software. Two 96 well plates were prepared with 200 μL of a 10% solution of RBC in culture medium. After sorting, individual infected cells were deposited into 96 well culture plates prepared with 200 μL of a 10% solution of RBC in culture medium, and cultured in a 3% oxygen atmosphere. Screening of individual culture wells for parasite DNA was performed using PCR with RAP (BoN-F and Bon-R) primers. Positive wells were transferred to a 48 well plate and RNA and protein collected for expression analysis.

### Experimental infection of calves

Holstein calves were obtained at 3–6 months of age from a Washington State dairy. Animal procedures were approved by the University of Idaho Animal Care and Use Committee (#2013–66) in accordance with institutional guidelines based on the U.S. National Institutes of Health (NIH) Guide for the Care and Use of Laboratory Animals.

Hereford calves obtained at seven-month old of age were acquired from a naturally tick-free area, housed in individual tick-proof pens on slatted floors and maintained at the Faculdade de Veterinária, Universidade Federal do Rio Grande do Sul, Brazil. Animal care was in accordance with institutional guidelines. Animal procedures were approved by the Universidade Federal do Rio Grande do Sul ethics comitee (#26247).

Two four to five months old spleen-intact Holstein calves (bovine 1 and 2 –b1 and b2) were experimentally infected with cultured *B*. *bovis* parasites of the clonal parasite line HlGST-Cln and one age-matched Holstein calf (bovine 3- b3) was experimentally infected with the parasite line control GFP-Cln. All experimentally infected animals were infected with 5×10^7^ infected erythrocytes, in a total volume of 3 mL, via intravenous route. All animals were monitored for signs of acute babesiosis: parasitemia, fever and hematocrit. Blood samples were collected daily after the infection for DNA extraction to monitor infection by PCR. Seven days after inoculation 250 mL of blood were collected, defibrinated and cultured in a 48 well culture plate, in a 3% oxygen atmosphere at 37°C, for recovery of parasites from the blood of infected animals.

Two weeks after immunization serum samples were collected to detect the presence of anti-GST or anti-MSA antibodies, using western blot (as described in expression analysis). A cELISA for the detection of *B*. *bovis* anti RAP-1 antibodies was performed using a kit provided by VMRD (Pullman, WA) on serum samples, as previously described [47,48]. cELISA was performed as described in [48]. 5ng of RAP-1 antigen was used for plate coating. Antigen-coated plates were blocked with PBS plus 0.2% Tween 20 containing 20% nonfat dry milk for 1 h at room temperature, followed by 100 μl of test sera in duplicate wells for 30 min. After the serum from each well was removed, 100 μl (50 ng/well) of BABB75A4 MAb was added, and the plates were incubated at room temperature for 15 min. The percent inhibition of the mean of test sample wells was computed as follows: 100 − [(the OD of the test sample/the mean OD of the normal control serum panel) × 100].

For the second animal trial involving tick challenge, six seven-month old Hereford animals were experimentally infected with 5×10^7^ infected erythrocytes, in a total volume of 3 mL, using the intravenous route. These calves were randomly divided into two groups of three test (HlGST-Cln parasites–B1, B2 and B3) and three control (GFP parasites–B4, B5 and B6) animals. The calves were monitored for signs of acute babesiosis including parasitemia, rectal fever, hematocrit and fibrinogen, daily, during 10 days after immunization, and also prior to the inoculation to check basal levels. All animals were also tested for serological levels of creatinine, urea, aspartate aminotransferase, alkaline phosphatase, albumin, total proteins and globulins, and a complete hemogram panel. Physiological data was statistically analyzed using repeated-measures analysis of variance with a post hoc Tukey-Kramer. Blood and serum samples were collected before immunization and 5 and 10 days after the inoculation. Levels of GST-specific antibodies in the serum samples were assessed by dot-blot. Nitrocellulose membrane circles were coated with 3 μg of recombinant HlGST antigen. The membranes were dried and incubated for 1 hour with a 2.5% skim milk in PBST blocking solution prior to probing with sera from B1-B6 animals at a 1:10 dilution for 16h. Anti-IgG alkaline phosphatase (Sigma) conjugate was used as secondary antibodies and the results were visualized using NBT (Fermentas) and BCIP (Fermentas). Antibody binding was evaluated by membrane scanning using software Image J and used to compare the difference among pre-immune (day 1) and post-immunization (day 30) cattle sera from vaccinated and control groups. Color intensity difference data was statistically analyzed using repeated-measures analysis of variance with a post hoc Tukey-Kramer.

Thirty days after immunization, all six calves were infested with approximately 20,000 10-day-old tick larva (from 1g of *R*. *microplus* Porto Alegre strain hatched eggs) placed on the dorsal region of each calf. From day 20 after infestation until the end of adult tick feeding period, all tick females that had dropped from the host were collected, counted and weighed daily. A total of 5 g of engorged adult female ticks from each animal, per day, were kept in petri dishes at 28°C and 85% relative humidity to evaluate oviposition, through the calculation of egg laying capacity, egg hatching and calculation of egg fertility. Egg laying capacity was obtained by calculating the ratio between total weight of females placed for egg laying and the total weight of resultant eggs. Egg fertility was calculated as the ratio between total egg weight and weight of hatched larvae from those eggs. All data collected after infestation was analyzed using standard *t*-test.

## Supporting Information

S1 Fig Bidirectional promoter test in stable transfection procedure. A) Schematic representation of the dual reporter plasmid *pEf-eGFP-RFP-BSD* which was generated using the *pMSASignal-HlGST-GFP-BSD* plasmid as backbone. The B, C, and D boxes show the same field in a fluorescence microscope of transfected parasites using different filters. B) Red filter, showing that the parasites are inside of RBC. C) Red filter, with less light, showing the red fluorescent parasites. D) Green filter, showing a reduced amount of green fluorescent parasites in comparison to red fluorescent ones.
(TIF)

S2 Fig Screening of *in vitro* cultures derived from FACS separated cells. Plates 1 and 2 depict **s**creening of clonal lines derived from the parasite line HlGST by FACs. The screening was performed by PCR amplification of a fragment derived from the *B*. *bovis RAP-1* gene. The eight *RAP-1* positive culture wells, out of the total of 192 wells analyzed, are marked with #.
(TIF)

S3 Fig Characterization of recovered parasites from infected animals. Panel A: RT-PCR amplifications designed for the detection of HlGST, GFP and RAP transcripts. Lane 1: HlGST-Cln recovered from b1. Lane 2: HlGST-Cln recovered from b2. Lane 3: GFP-Cln recovered from b3. Lane 4: not-transfected *B*. *bovis* control. Lane 5: *pMSASignal-HlGST-GFP-BSD* plasmid. Lane 6:*pGFP/BSD/EF* GFP plasmid. Lane 7: negative control. B) Western blot using rabbit serum anti-HlGST to confirm HlGST expression by recovered parasites. Anti-GFP antibody and anti MSA were also used. Lane 1: HlGST-Cln recovered from b1. Lane 2: HlGST-Cln recovered from b2. Lane 3: GFP-Cln recovered from b3. Lane 4: not-transfected *B*. *bovis* control. C) Agarose gel analysis of the PCR amplification products from integration PCR using the group of primers described in Fig 4 and genomic DNA as template. Lane 1: HlGST-Cln recovered from b1. Lane 2: HlGST-Cln recovered from b2. Lane 3: GFP-Cln recovered from b3. Lane 4: non-transfected *B*. *bovis* control. Lane 5: *pMSASignal-HlGST-GFP-BSD* plasmid. Lane 6:*pGFP/BSD/EF* GFP plasmid. Lane 7: negative control. D) Southern blot analysis performed on *B*. *bovis* gDNA using HlGST and GFP probes. Lane 1: HlGST-Cln recovered from b1. Lane 2: HlGST-Cln recovered from b2. Lane 3: GFP-Cln recovered from b3. Lane 4: not-transfected *B*. *bovis* control. Lane 5: *pMSASignal-HlGST-GFP-BSD* plasmid. Lane 6:*pGFP/BSD/EF* GFP plasmid.
(TIF)

S4 Fig Clinical responses of calves to vaccination. Graphics presenting hematocrit (Panel A), temperature (Panel B) and fibrinogen (Panel C) of animals vaccinated with HlGST-Cln (Bovines 1, 2 and 3) or GFP-Cln (Bovine 4,5 and 6). Data collected previously and 10 days after vaccination.
(TIF)

S5 Fig Anti-GST response in calves during second animal trial vaccination. Previously to tick challenge, animals were tested for the presence of anti-HlGST antibodies. Upper panel show dot blot assay result. Pre-immune and 30 day serum were probed against HlGST, and only HlGST-Cln vaccinated animals presented reaction (B1, B2 and B3). The graphics represent the the densitometric data obtained from the same assay showing that there is a statistical difference among immunized groups in response to HlGST recognition. Positive control is a bovine serum of an animal immunized 3 times with recombinant protein. *Statistically significant (p < 0.01)
(TIF)

S1 Table Biochemical parameters from immunized bovines. Bovines 1 to 6 vaccinated with the GST-Cln (Bovines 1, 2 and 3) GFP-Cln (Bovine 4, 5 and 6) parasites.
(TIF)

S2 Table Hematological parameters from immunized bovines. Bovines 1 to 6 vaccinated with the GST-Cln (Bovines 1, 2 and 3) GFP-Cln (Bovine 4, 5 and 6) parasites
(TIF)

S1 Video Video showing the erythrocyte evasion process by *B*. *bovis* parasites. Expression of the reporter gene *GFP* was analyzed by fluorescence analysis using an Axioskop 40 fluorescent microscope (Zeiss Micro Imaging), connected to an Axiocam MR camera for image acquisition.
(MP4)

S1 File Transfection of *B*. *b*ovis parasites using a dual reporter plasmid. (PDF)
