# Supplementary figures and images for: Transfected Babesia bovis Expressing a Tick GST as a Live Vector Vaccine
Source: PLoS Negl Trop Dis. 2016 Dec 2;10(12):e0005152. doi: 10.1371/journal.pntd.0005152 (PMC5135042; doi:10.1371/journal.pntd.0005152)

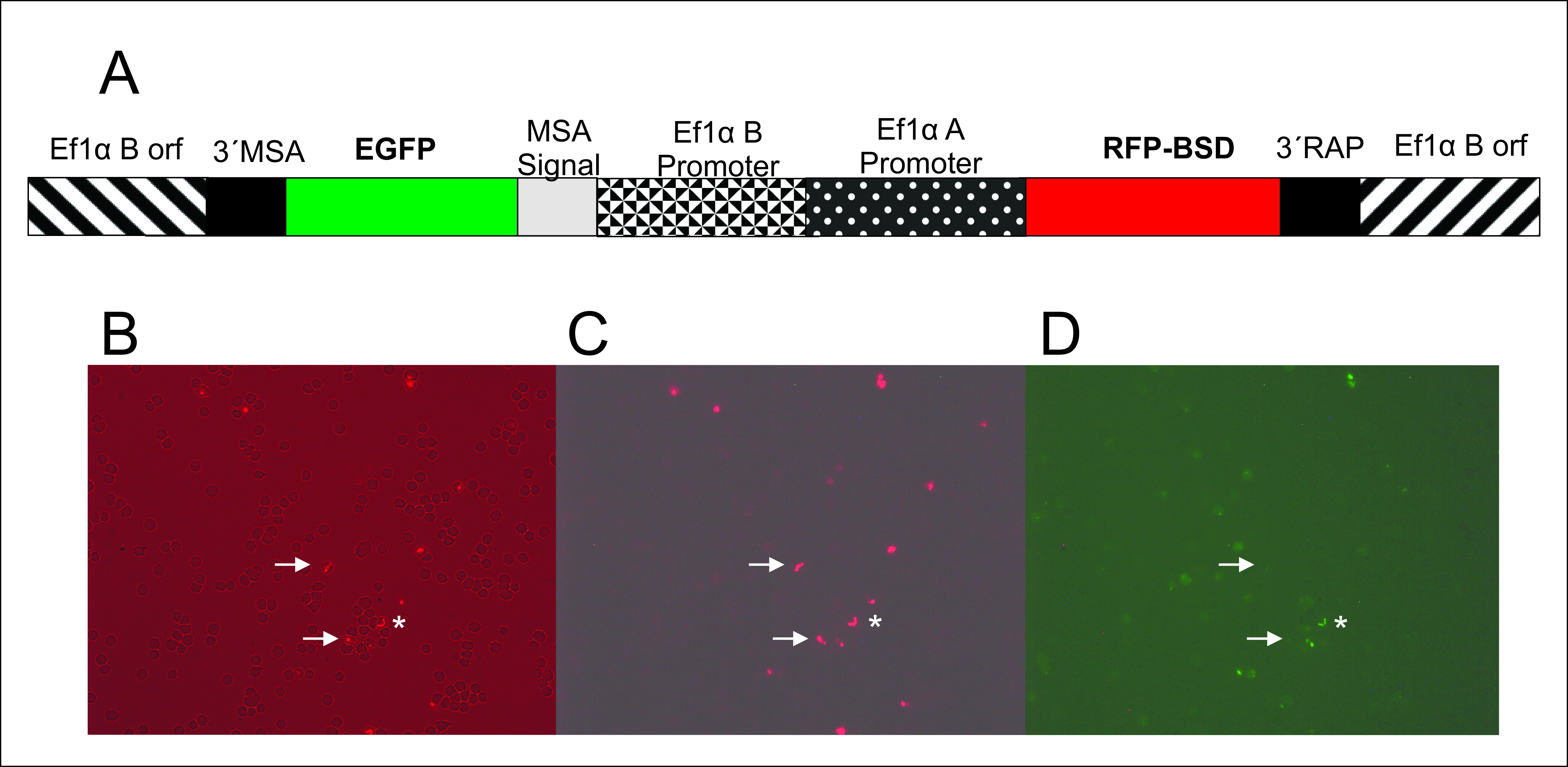

Supplement: S1 Fig — A) Schematic representation of the dual reporter plasmid pEf-eGFP-RFP-BSD which was generated using the pMSASignal-HlGST-GFP-BSD plasmid as backbone. The B, C, and D boxes show the same field in a fluorescence microscope of transfected parasites using different filters. B) Red filter, showing that the parasites are inside of RBC. C) Red filter, with less light, showing the red fluorescent parasites. D) Green filter, showing a reduced amount of green fluorescent parasites in comparison to red fluorescent ones. (TIF) [file pntd.0005152.s001.tif]

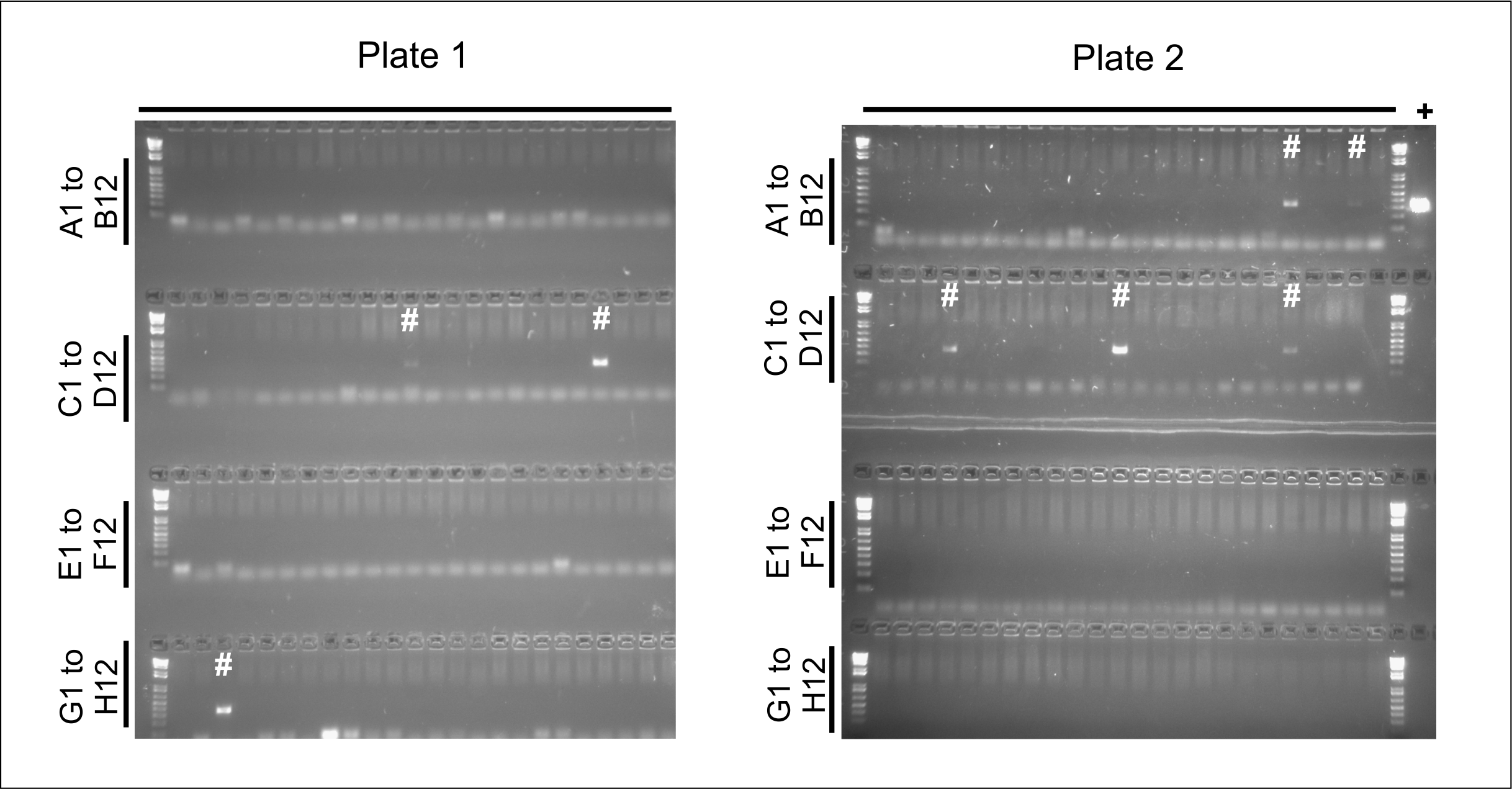

Supplement: S2 Fig — Plates 1 and 2 depict screening of clonal lines derived from the parasite line HlGST by FACs. The screening was performed by PCR amplification of a fragment derived from the B. bovis RAP-1 gene. The eight RAP-1 positive culture wells, out of the total of 192 wells analyzed, are marked with #. (TIF) [file pntd.0005152.s002.tif]

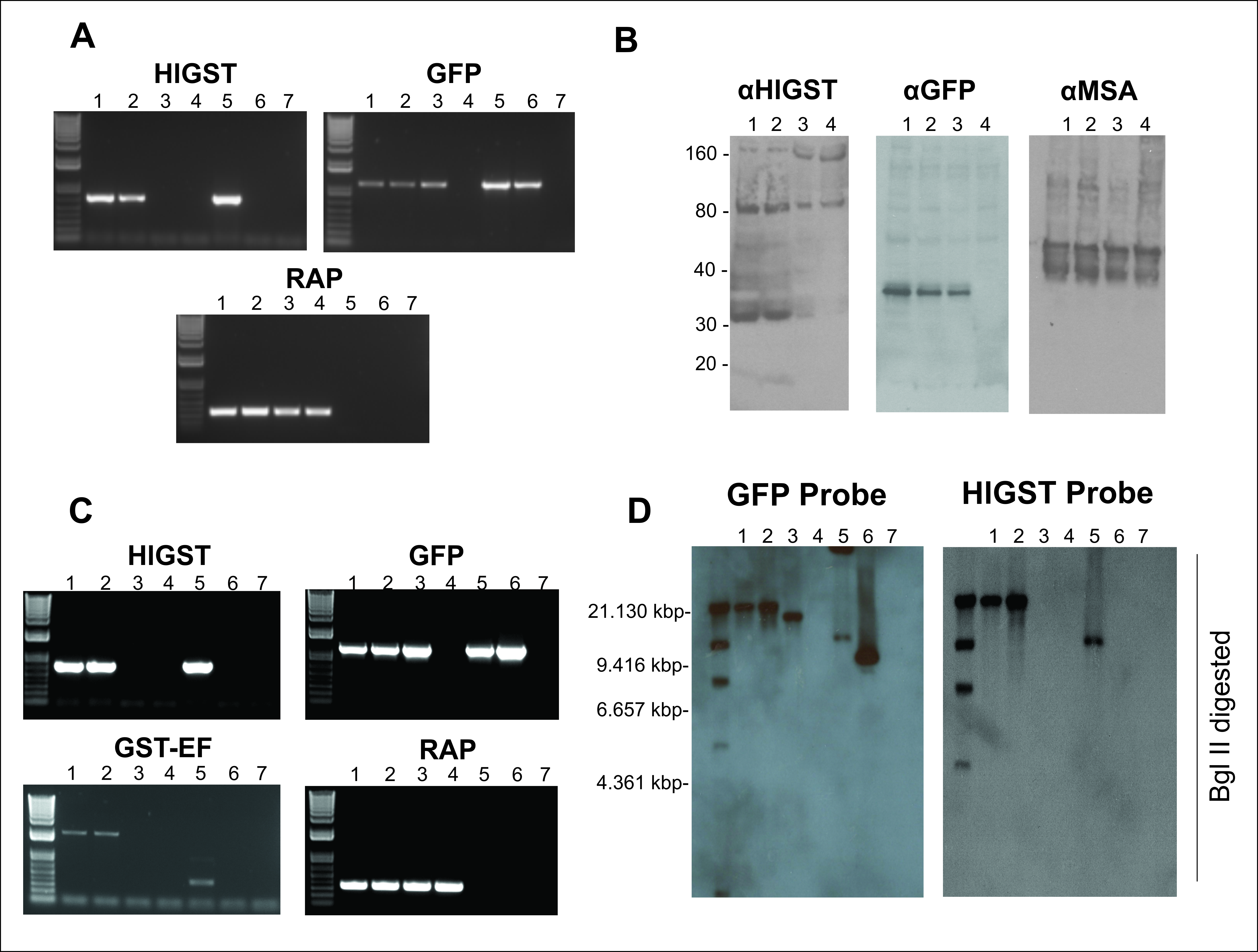

Supplement: S3 Fig — Panel A: RT-PCR amplifications designed for the detection of HlGST, GFP and RAP transcripts. Lane 1: HlGST-Cln recovered from b1. Lane 2: HlGST-Cln recovered from b2. Lane 3: GFP-Cln recovered from b3. Lane 4: not-transfected B. bovis control. Lane 5: pMSASignal-HlGST-GFP-BSD plasmid. Lane 6:pGFP/BSD/EF GFP plasmid. Lane 7: negative control. B) Western blot using rabbit serum anti-HlGST to confirm HlGST expression by recovered parasites. Anti-GFP antibody and anti MSA were also used. Lane 1: HlGST-Cln recovered from b1. Lane 2: HlGST-Cln recovered from b2. Lane 3: GFP-Cln recovered from b3. Lane 4: not-transfected B. bovis control. C) Agarose gel analysis of the PCR amplification products from integration PCR using the group of primers described in Fig 4 and genomic DNA as template. Lane 1: HlGST-Cln recovered from b1. Lane 2: HlGST-Cln recovered from b2. Lane 3: GFP-Cln recovered from b3. Lane 4: non-transfected B. bovis control. Lane 5: pMSASignal-HlGST-GFP-BSD plasmid. Lane 6:pGFP/BSD/EF GFP plasmid. Lane 7: negative control. D) Southern blot analysis performed on B. bovis gDNA using HlGST and GFP probes. Lane 1: HlGST-Cln recovered from b1. Lane 2: HlGST-Cln recovered from b2. Lane 3: GFP-Cln recovered from b3. Lane 4: not-transfected B. bovis control. Lane 5: pMSASignal-HlGST-GFP-BSD plasmid. Lane 6:pGFP/BSD/EF GFP plasmid. (TIF) [file pntd.0005152.s003.tif]

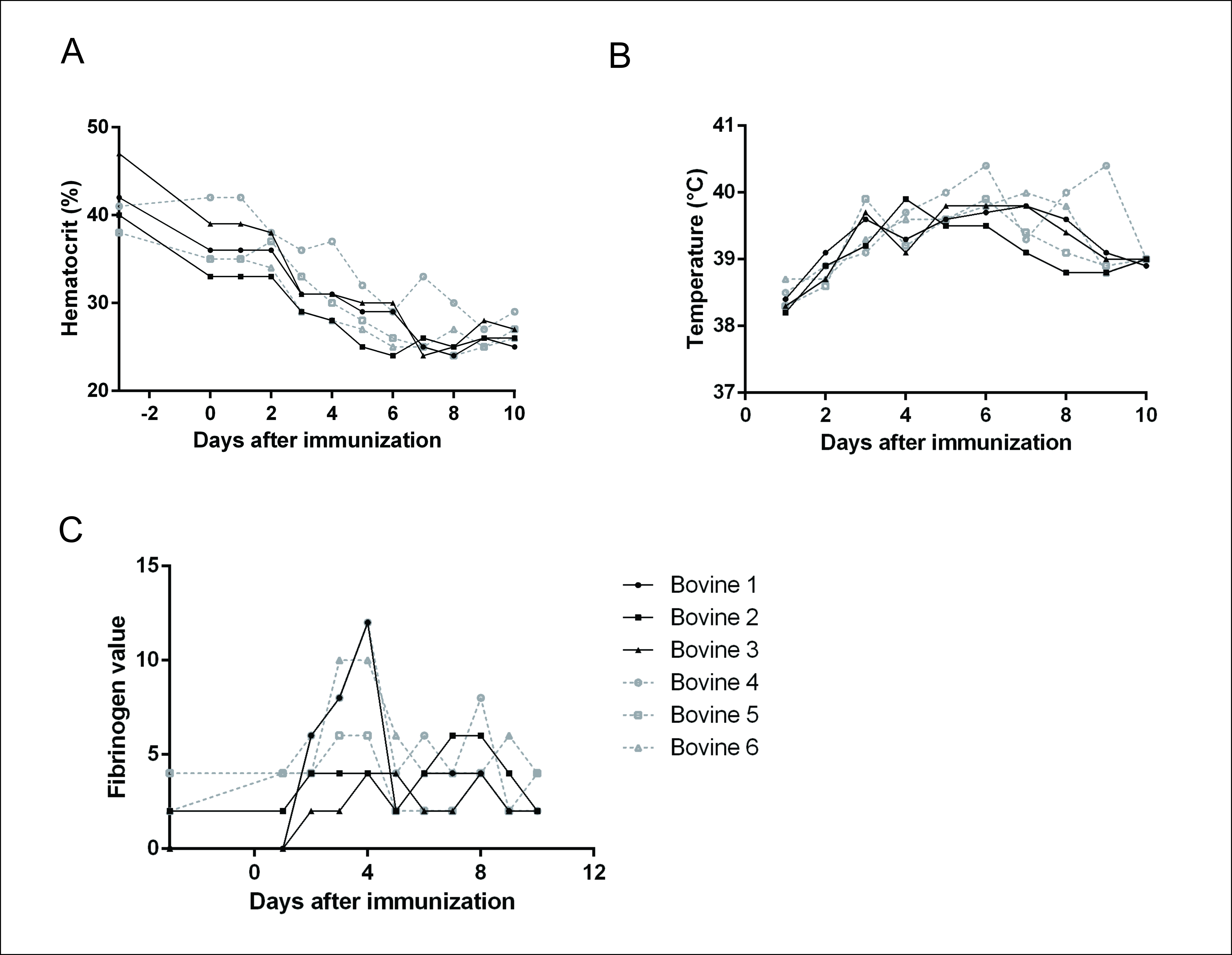

Supplement: S4 Fig — Graphics presenting hematocrit (Panel A), temperature (Panel B) and fibrinogen (Panel C) of animals vaccinated with HlGST-Cln (Bovines 1, 2 and 3) or GFP-Cln (Bovine 4,5 and 6). Data collected previously and 10 days after vaccination. (TIF) [file pntd.0005152.s004.tif]

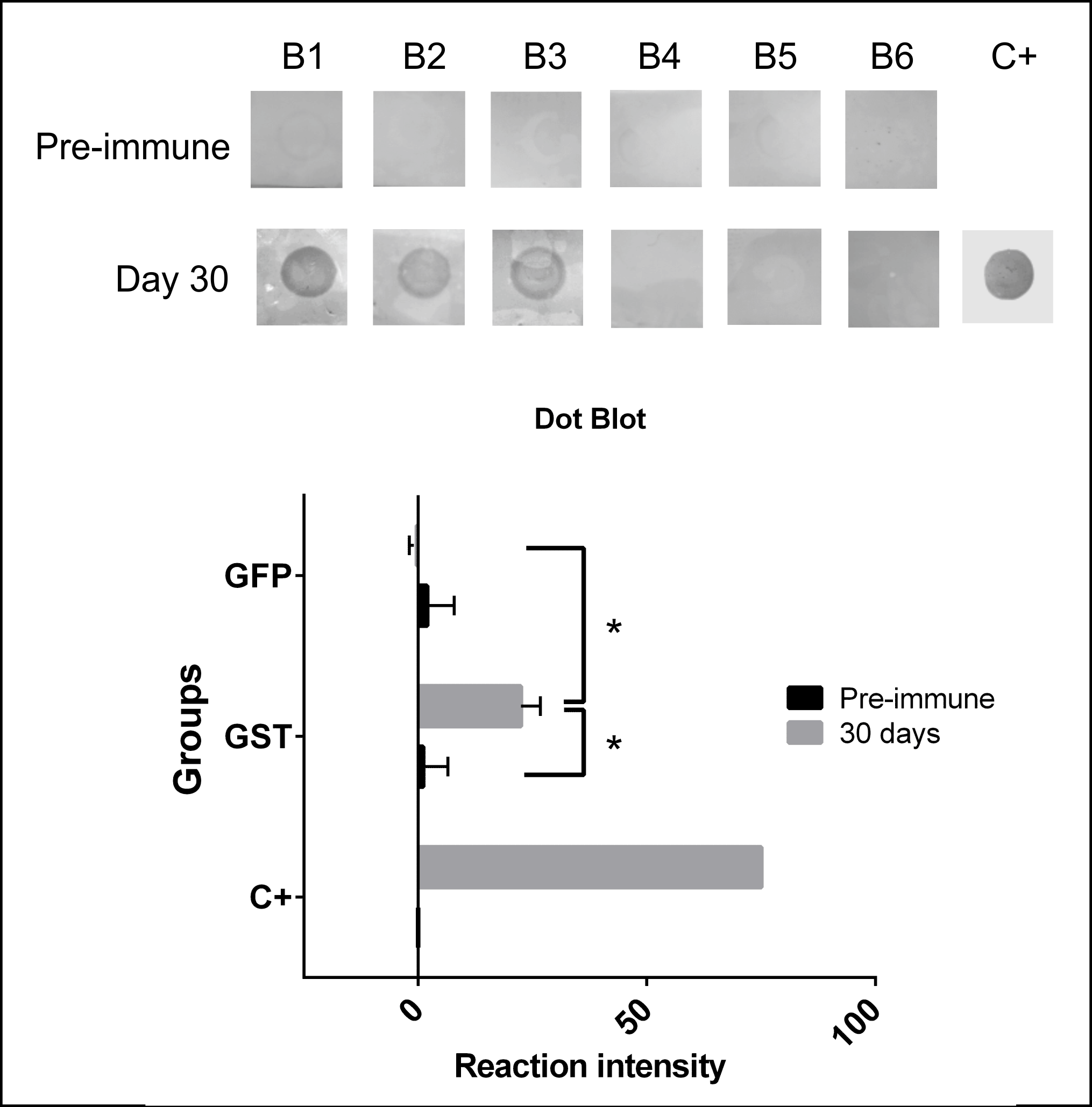

Supplement: S5 Fig — Previously to tick challenge, animals were tested for the presence of anti-HlGST antibodies. Upper panel show dot blot assay result. Pre-immune and 30 day serum were probed against HlGST, and only HlGST-Cln vaccinated animals presented reaction (B1, B2 and B3). The graphics represent the the densitometric data obtained from the same assay showing that there is a statistical difference among immunized groups in response to HlGST recognition. Positive control is a bovine serum of an animal immunized 3 times with recombinant protein. *Statistically significant (p < 0.01) (TIF) [file pntd.0005152.s005.tif]

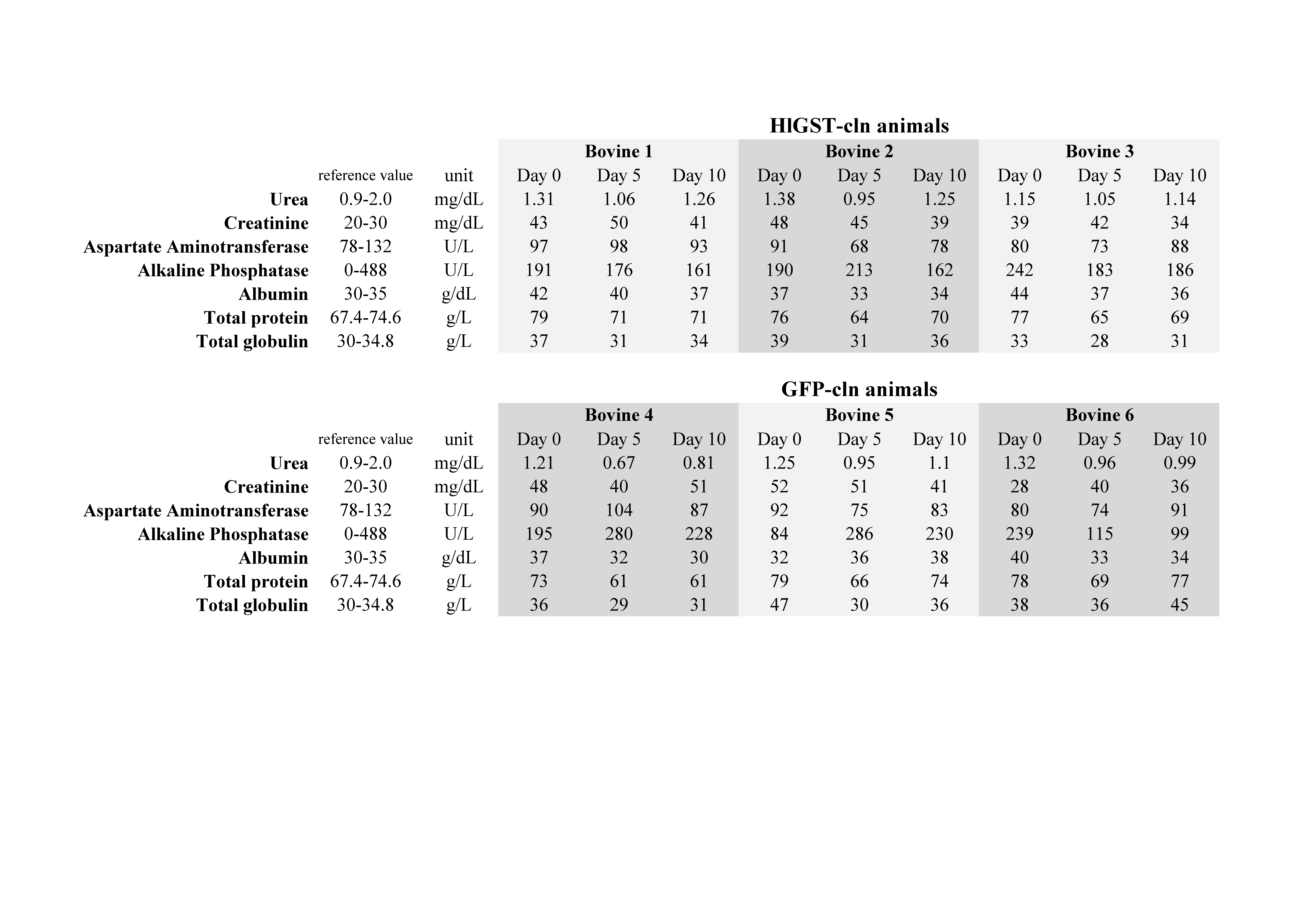

Supplement: S1 Table — Bovines 1 to 6 vaccinated with the GST-Cln (Bovines 1, 2 and 3) GFP-Cln (Bovine 4, 5 and 6) parasites. (TIF) [file pntd.0005152.s006.tif]

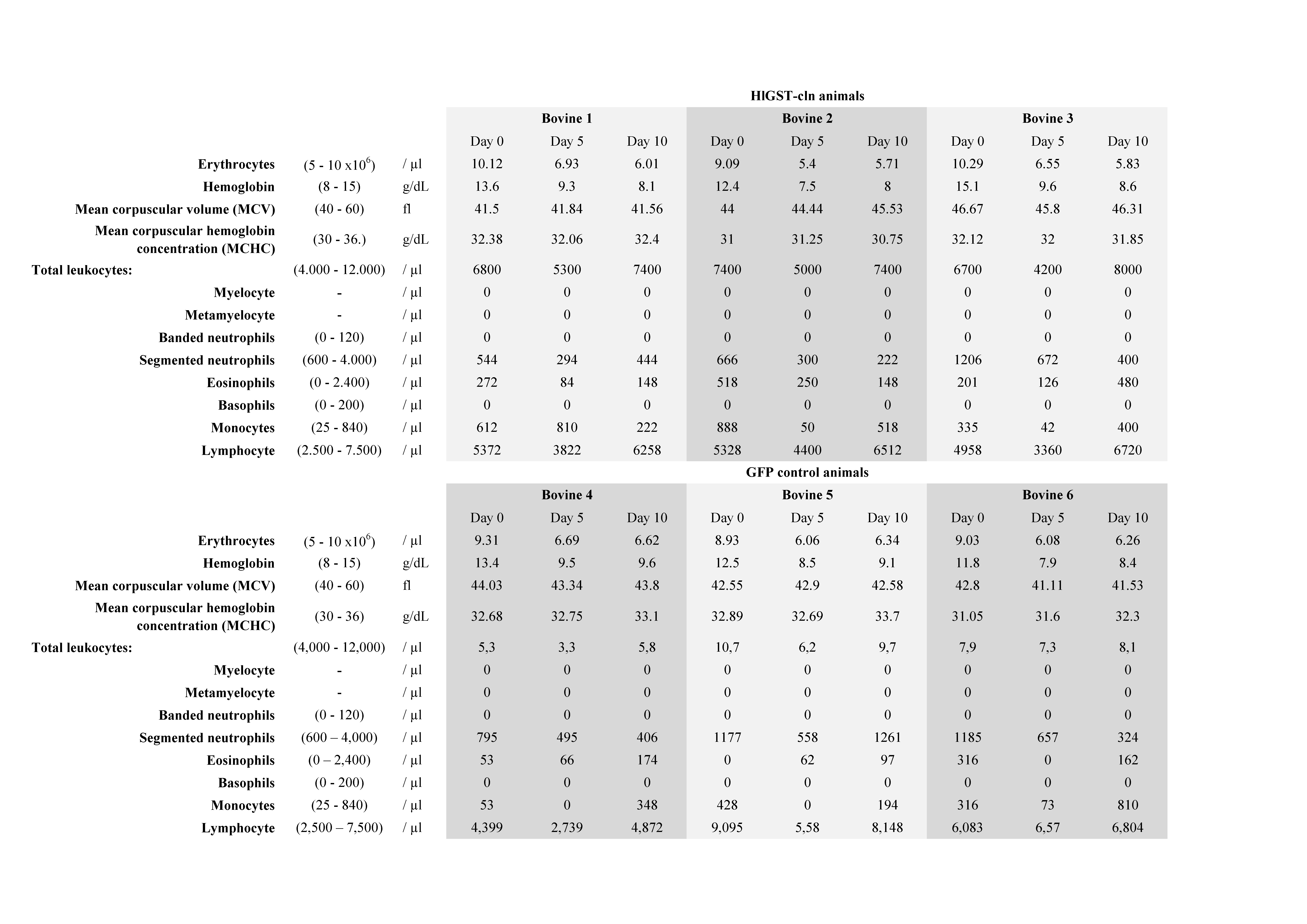

Supplement: S2 Table — Bovines 1 to 6 vaccinated with the GST-Cln (Bovines 1, 2 and 3) GFP-Cln (Bovine 4, 5 and 6) parasites (TIF) [file pntd.0005152.s007.tif]
