# Supplementary material for: Transfected Babesia bovis Expressing a Tick GST as a Live Vector Vaccine
Source: PLoS Negl Trop Dis. 2016 Dec 2;10(12):e0005152. doi: 10.1371/journal.pntd.0005152 (PMC5135042; doi:10.1371/journal.pntd.0005152)
Supplement: S1 File — (PDF) [file pntd.0005152.s009.pdf]

S1 File: Transfection of *B. bovis* parasites using a dual reporter plasmid.

We conducted experiments aimed at testing the homogeneity of the stably transfected parasite population and the efficiency of the selected dual promoter to control expression of two distinct fluorescent proteins by single transfected parasites. To this end, a stable transfection plasmid construct denominated *pEf-eGFP-RFP-BSD* containing the reporter genes eGFP and RFP under the control of the dual ef-1a promoter was generated (S1A Fig), as shortly described in S1 File, and transfected into *B. bovis* T3B parasites. Viable intracellular blasticidin resistant and fluorescent parasites were observed twenty days after electroporation (S1B, C and D Figs). Fluorescence microscopy analysis demonstrated the occurrence of both, double and single fluorescent transfected parasites in the blasticidin selected cultures (S1B, C and D Figs). Because dual green and red fluorescence was not detected in all transfected parasites, we concluded from these experiments that the cultured blasticidin resistant transfected parasites that emerged in the blasticidin selected cultures are a mix of heterogeneous parasite population. These results can be explained by possible differential dual promoter activity, or by the occurrence of different patterns of insertion of the transfection construct in the transfected parasites. Overall, the data suggested at least two alternative patterns of insertion of the transfect construct by homologous recombination, with one of the patterns excluding the insertion of one of the transfected genes. Because the general architecture of both *pEf-eGFP-RFP-BSD* and *pMSASignal-HlGST-GFP-BSD* is similar, it can be predicted that transfection with plasmid *pMSASignal-HlGST-GFP-BSD* will also result in a similar heterogeneous population of transfected parasites. The findings supported the need for the selection of a clonal line of parasites with dual transfected gene expression from the mixed population for further studies.
